# Simultaneous regression and classification for drug sensitivity prediction using an advanced random forest method

**DOI:** 10.1038/s41598-022-17609-x

**Published:** 2022-08-05

**Authors:** Kerstin Lenhof, Lea Eckhart, Nico Gerstner, Tim Kehl, Hans-Peter Lenhof

**Affiliations:** Center for Bioinformatics, Saarland University, Saarland Informatics Campus (E2.1), 66123 Saarbrücken, Saarland Germany

**Keywords:** Machine learning, Computational models, Cancer therapy, Tumour heterogeneity

## Abstract

Machine learning methods trained on cancer cell line panels are intensively studied for the prediction of optimal anti-cancer therapies. While classification approaches distinguish effective from ineffective drugs, regression approaches aim to quantify the degree of drug effectiveness. However, the high specificity of most anti-cancer drugs induces a skewed distribution of drug response values in favor of the more drug-resistant cell lines, negatively affecting the classification performance (class imbalance) and regression performance (regression imbalance) for the sensitive cell lines. Here, we present a novel approach called SimultAneoUs Regression and classificatiON Random Forests (SAURON-RF) based on the idea of performing a joint regression and classification analysis. We demonstrate that SAURON-RF improves the classification and regression performance for the sensitive cell lines at the expense of a moderate loss for the resistant ones. Furthermore, our results show that simultaneous classification and regression can be superior to regression or classification alone.

## Introduction

A major goal of personalized medicine is to tailor treatment to patients based on their specific genetic, phenotypic, and molecular characteristics. Especially in cancer, treatment customization can be challenging since the (epi)genetic profile of cancer cells varies considerably. This heterogeneity has a substantial impact on the efficacy of drugs.

Various machine learning (ML) approaches have been developed to aid in therapy optimization and elucidate the relationships between the (epi)genomic characteristics of cancer cells and the treatment outcome. In particular, classification approaches have been utilized to help identify tumors that may benefit from treatment with particular drugs or their combinations, while regression approaches were designed to quantify the tumors’ degree of sensitivity^1,2^. A putative approach to integrating ML methods into the optimization of tumor treatment would be first to distinguish effective from ineffective drugs by classification and then prioritize the effective ones by their efficiency, toxicity, etc., by regression. Our results show that a combined regression and classification approach can be superior to regression or classification alone.

To develop ML models, cancer cell line panels are often used^3–8^. These panels are supposed to mirror tumor traits to a considerable extent. One of the largest publicly available panels containing high-dimensional multi-omics measurements as well as drug sensitivity profiles of cell lines is the Genomics of Drug Sensitivity in Cancer (GDSC)^4^. Here, a continuous drug sensitivity measure, e.g., IC50 or AUC, quantifies the cell line sensitivity to a drug. Based on these values, the cell lines can also be partitioned into classes such as sensitive and resistant.

ML regression and classification methods have been successfully applied to such cell line panels in supervised learning settings using the molecular characteristics of the cell lines as features and the sensitivity of the cell lines to drugs as response variables^4–18^. The DREAM7 challenge, for example, evaluated a wide range of methodologies, including linear models, kernel methods, and tree-based methods^5^. Neural networks have also become popular for estimating the sensitivity of cancer cell lines to anti-cancer drugs^9,10^. Methods such as MOCA^11^, LOBICO^12^, or MERIDA^13^ perform classification by employing optimization techniques and thereby learn well-interpretable Boolean rules from binarized omics-feature sets. All mentioned approaches predict the sensitivity for single drugs. However, monotherapy can promote the development of drug resistances. Hence, there also exist methods that predict the sensitivity for drug combinations using cancer cell line panels^1,2,19,20^.

A general issue with drug sensitivity data is that the high specificity of (targeted) anti-cancer drugs leads to an underrepresentation of sensitive samples. In the GDSC, for instance, the sensitive cell lines are heavily underrepresented with an average sensitive-to-resistant ratio of 1:10 per drug^13^. Consequently, the corresponding classification performance in terms of statistical sensitivity is often poor. However, it is of utmost importance for personalized medicine to identify the sensitive tumors, and classification methods trained on cancer cell line panels usually address this issue specifically^12,13^.

In this paper, we demonstrate that for regression, a related issue, known as regression imbalance^21^, occurs for a multitude of ML methods. While these methods tend to perform well for the prediction of the drug sensitivity values around the mean drug response of the cell lines, they systematically over- or under-estimate the upper and lower end of the prediction scale (cf. Fig. 2). However, for medical decision making, especially the lower (highly sensitive) end of the scale is of particular interest, whereas the upper end is of less importance and a correct identification of drug-resistance would be sufficient. The described class imbalance usually aggravates the prediction problem for the sensitive samples since the mean is shifted towards the resistant ones. Especially for the most sensitive samples, the predictive performance deteriorates enormously. In literature, the tendency to predominantly predict values around the mean was already described for random forests (RFs)^22,23^. An approach that can be used to address this issue is HARF (Heterogeneity-Aware Random Forests) proposed by Rahman et al.^24^. By integrating cancer types as class information into a conventional RF regressor, Rahman et al. perform a cancer type prediction and use it to weight the trees for the regression. As a consequence, they achieve predictions around the mean of each cancer type. For each drug, Rahman et al. typically consider only cancer types with sufficiently different average drug responses and neglect the other cancer types. In practice, they usually resort to the investigation of two cancer types. Consequently, they dismiss large portions of the data.

**Figure 1:**
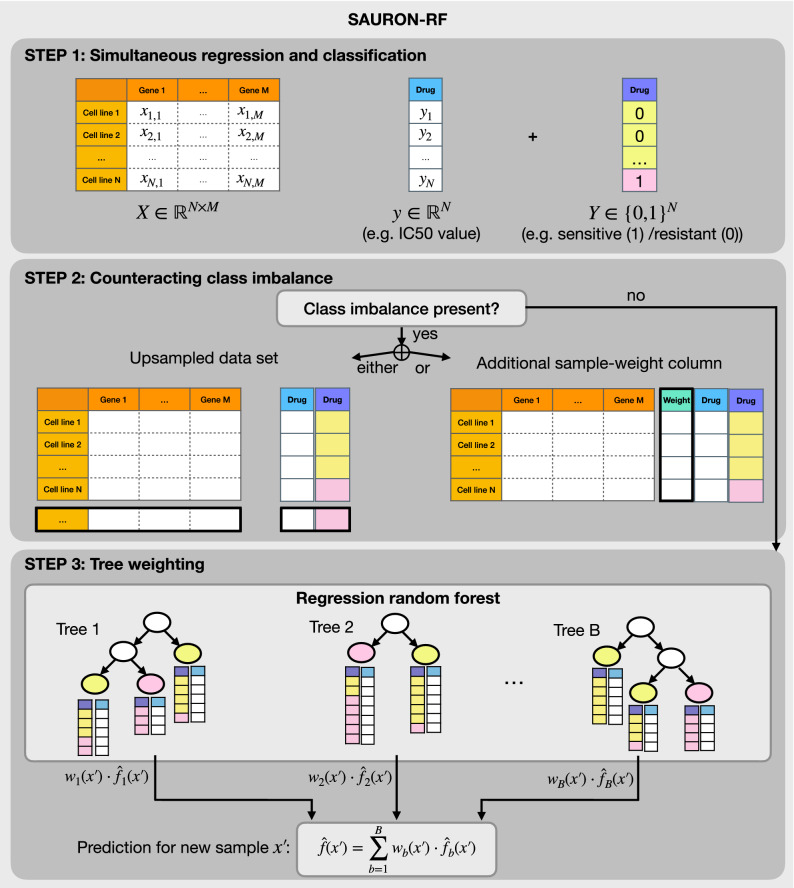
Workflow SAURON-RF. The figure depicts the three-step workflow of SAURON-RF. The SAURON-RF procedure starts with a preprocessing phase, in which the dimensionality of the input gene expression matrix is reduced, and the binary drug response (sensitive/resistant) is derived from the logarithmized IC50 values. In the second step, SAURON-RF addresses class imbalance by either performing upsampling or using sample-specific weights. In the last step, a regression random forest is trained from the given continuous IC50 values and the gene expression matrix. Then, each leaf node is assigned to the majority class of its corresponding samples. Thereby, the regression random forest can also be used to classify samples by majority vote over the trees. For a new sample $x'$, a continuous prediction is calculated by multiplying the tree-specific weights $w_b(x')$with the tree-specific predictions ${\hat{f}}_b(x')$.

To address the class and regression imbalance problem for drug sensitivity prediction, we developed a novel approach called SimultAneoUs Regression and classificatiON Random Forests (SAURON-RF), which is based on the idea of performing a joint regression and classification analysis with RFs similar to the HARF approach. However, we propose several RF extensions that aim to address the regression and class imbalance problem directly. The three main extensions are (cf. Fig. 1):

1. We propose a partition into sensitive and resistant cell lines as class assignment for SAURON-RF instead of cancer types. Thus, SAURON-RF inherently meets the demand for distinct average drug responses per class without the disadvantage of sample exclusion.
2. We suggest counteracting regression and class imbalance by using either upsampling techniques or calculating sample-specific weights.
3. We introduce alternatives to the tree weighting scheme by Rahman et al. to improve the regression performance.

We conducted a comprehensive study on the GDSC data set to evaluate our novel approach. We demonstrate that SAURON-RF outperforms a diverse set of regression and classification RFs including HARF. In particular, the statistical sensitivity and the regression performance for the sensitive cell lines improve considerably at the cost of a moderate effect on the resistant cell lines.

## Results

To address the drug sensitivity prediction problem, we developed the RF-based method SAURON-RF. In the following sections, we first introduce the data sets used to study ML methods, including SAURON-RF. Then we show how regression and class imbalance affect the performance of ML methods. Finally, we present SAURON-RF and compare its performance to various other RF-based methods.

### Data sets and analysis set-up

For our analyses, we employ data from the GDSC database. We downloaded the gene expression and the drug screen information in the form of IC50 values from the GDSC website. We only trained models for drugs with more than 750 available cell lines, resulting in 86 investigated drugs. Since we perform a joint regression and classification analysis, we also derive a discretized drug response from the continuous IC50 values by applying a procedure introduced by Knijnenburg et al.^12^. Briefly, we obtain a division into sensitive and resistant cell lines for each drug by comparing the continuous IC50 values to a drug-specific threshold. For a detailed description of the data set compilation, please refer to Methods.

Besides training models for our own method SAURON-RF, we trained models for elastic net, neural networks, boosting trees, random forests, and the HARF method. We assess the quality of all models as follows: For each compound, we divide the data into a training (80%) and a test set (20%). We perform a 5-fold cross validation (CV) on the training set that we use to select the best hyperparameter combination for each method. A final model for each method is built on the complete training data set and applied to the test data set.

Since the gene expression data is very high-dimensional, we incorporate a heuristic feature selection procedure into model training for all methods. In summary, gene expression features are selected based on a trade-off between the mutual information (MI) of gene-drug and gene-gene pairs. In particular, we try to maximize the gene-drug (feature-response) MI while minimizing the gene-gene (feature-feature) MI, which is generally known as minimum-redundancy-maximum-relevance principle^25^. We investigated the performance for the following numbers of gene expression features: 20, 40, 60, 80 and 100. A more detailed description of the training process is provided in the Supplementary Material.

### SAURON-RF

**Figure 2:**
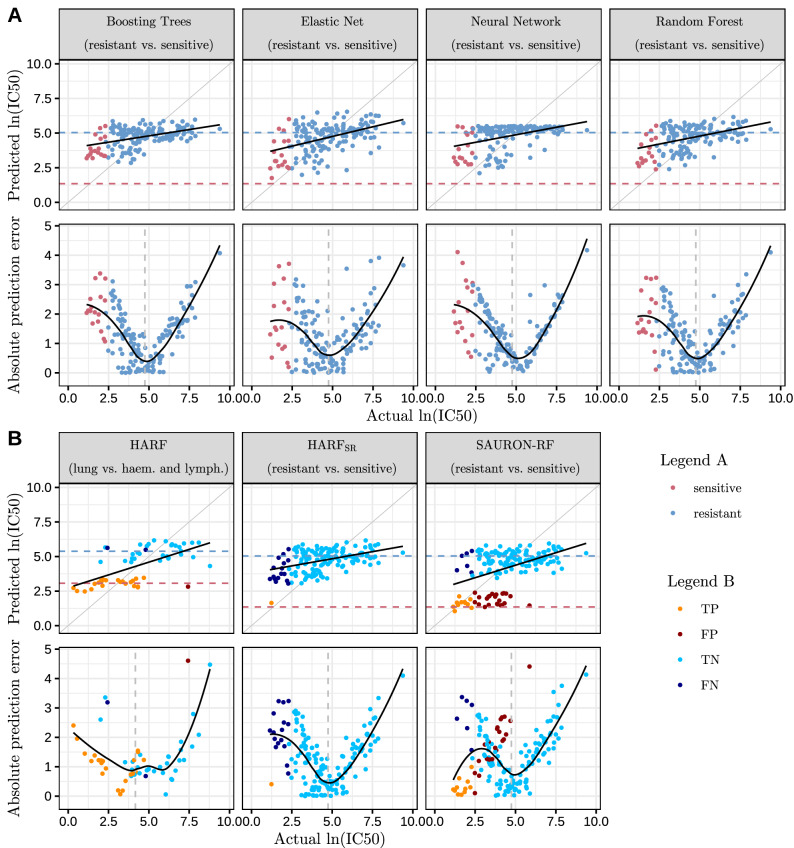
Regression performance of different ML methods. This figure exemplifies the performance of different ML algorithms when applied to the 5-Fluorouracil data set of the GDSC database using 20 input features. The upper rows of Fig. (**A**) and (**B**) show the predicted IC50 values plotted against the actual IC50 values including a fitted regression line, which is shown as a solid black line. The mean IC50 of training samples for each investigated class is depicted as a horizontal dashed line. The lower rows show the absolute prediction error. Here, the solid curve is a loess curve fitted to the error, the vertical dashed line gives the mean IC50 of all training samples. In Fig. (**A**), we compare boosting trees, elastic net, neural networks and random forests. The point coloring indicates the class assignment (sensitive or resistant). The first plot in Fig. (**B**) depicts the performance of the original HARF algorithm applied to a restricted version of this data set containing only cell lines from two cancer types with different average drug responses, i.e., haematopoietic/lymphoid cell lines and lung cell lines. The second plot shows the performance of HARF when applied to our proposed class division ($\text {HARF}_{SR}$), and the last plot depicts the performance of the suggested SAURON-RF algorithm (SAURON-RF simple s.w., binary sens t.w.). Here, the point coloring represents the classification performance, i.e. we depict true positives (TP), false positives (FP), true negatives (TN), and false negatives (FN).

We applied a multitude of ML regression methods, i.e., elastic net, neural networks, boosting trees, and random forests, to the 86 drugs from the GDSC data set and evaluated their performance. Expectedly, all tested approaches achieved a similar overall regression performance with elastic nets and random forests having a slightly superior average performance with respect to mean-squared error (MSE) and median squared error (cf. Supplementary Fig. 1). Moreover, we observed that they predict values around the mean response of the cell lines considerably well while the upper (highly resistant) and even more so the lower (highly sensitive) end of the sensitivity scale is mostly misfitted by all methods to a varying degree. In Fig. 2A, we show the performance of these methods for the drug 5-Fluorouracil, which is a typical example for a high class imbalance with 8.6% sensitive cell lines (cf. Supplementary Figs. 1–11 for further analyses).

#### Simultaneous regression and classification

We first addressed this issue for random forests by applying the HARF method, which performs a simultaneous classification and regression by integrating cancer types with distinct average drug responses into a random forest regressor. More specifically, Rahman et al. first train a conventional random forest regressor to predict the continuous drug sensitivity values for each drug. The trained forest can also be used for classification: Each leaf node can be assigned to the majority class of its training samples, and, hence, the whole forest can predict the class for a given sample based on a majority vote over all trees. For the sensitivity value prediction of the given sample, Rahman et al. use a *binary tree weighting* (binary t.w.) scheme, i.e., they only consider a tree if the predicted class of this tree is equal to the relative majority over all trees. For the final prediction of the sensitivity, the predictions of the selected trees are then averaged (cf. “Methods” for a more detailed description of HARF).

In Fig. 2B, we exemplary show a typical application scenario for HARF. We applied HARF to 5-Fluorouracil using two cancer types with distinct average drug responses. In particular, we opted for the two most abundant cancer types of the GDSC data set as classes: haematopoietic/lymphoid cell lines (139) and lung cell lines (135). When using these two cancer types, the resulting data set is very balanced. As expected, the HARF predictions for the haematopoietic/lymphoid cell lines and the lung cell lines are well-separated and concentrated around the mean of the respective class. However, there is still the tendency to misfit the upper and lower end of the prediction sale. Moreover, we had to exclude 532 of 806 available cell lines, which is especially critical in data poor settings.

#### New class definition

We suggest linking the regression and classification problems more closely by using the division into sensitive and resistant cell lines instead of cancer types. Consequently, we meet the demand for distinct average drug responses per class by definition and do not have to exclude samples. We call this method $\text {HARF}_{SR}$. In Fig. 2B, the performance of $\text {HARF}_{SR}$ is shown. As expected, the substantial class imbalance with only 8.6% of sensitive cell lines seems to negatively affect the classification performance of $\text {HARF}_{SR}$ since it identifies only one sensitive cell line correctly. Consequently, we do not obtain reasonable predictions for the sensitive cell lines.

#### Counteracting class imbalance

**Figure 3:**
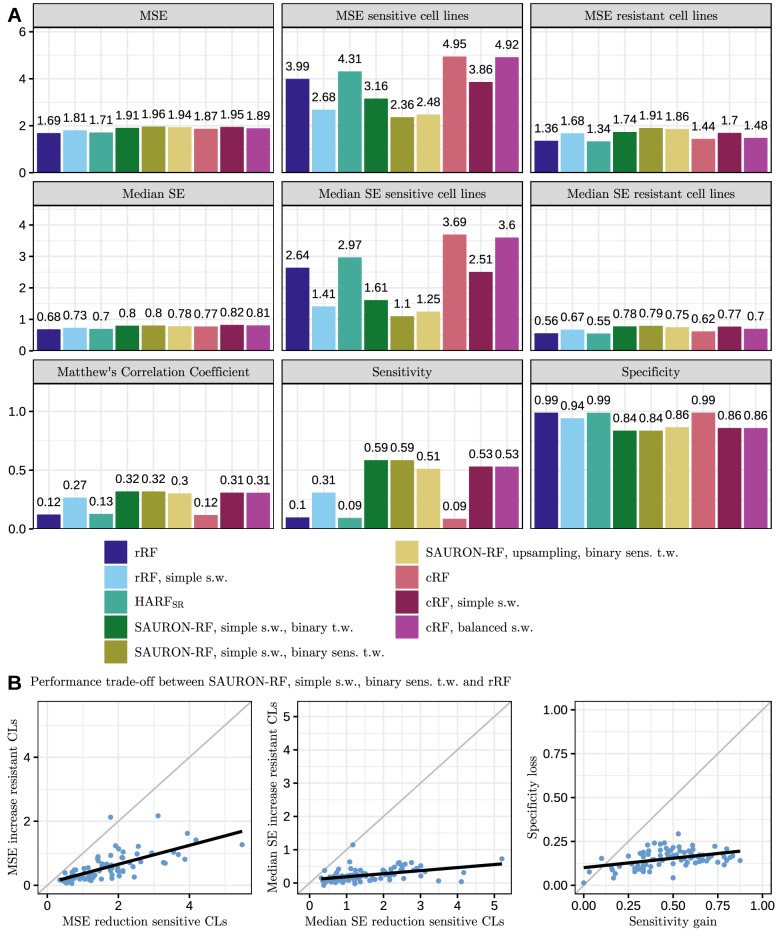
Random forest test set performance. In (**A**), we compare regression random forests (rRF), classification random forests (cRF), and $\text {HARF}_{SR}$ with different versions of our suggested approach SAURON-RF. We show the average test set performance across the 86 different drugs for 20 input features. Results for additional versions of SAURON-RF and other input feature set sizes can be found in the Supplementary Material (cf. Supplementary Figs. 12–18). In (**B**), we depict for each of the 86 drugs the tradeoff between the absolute reduction in test error for the sensitive cell lines and the absolute increase in test error for the resistant cell lines when comparing our best-performing version of SAURON-RF (SAURON-RF simple s.w., binary sens. t.w.) with rRF.

Class imbalance is a common obstacle for ML algorithms, especially for drug sensitivity prediction^13,26–30^. For most drug data sets in the GDSC, the sensitive cell lines are heavily underrepresented with an average sensitive-to-resistant ratio of 1:10. If we do not counteract class imbalance, the average sensitivity of classification random forests is only 9% (cf. Fig. 3).

To address the class imbalance, SAURON-RF performs either upsampling or uses sample-specific weights.

We suggest performing upsampling of the minority class (sensitive cell lines) prior to the training of the random forest. More specifically, we draw with replacement from the sensitive samples of a specific drug until the number of sensitive and resistant samples is equal. We refer to this upsampling scheme as *upsampling*.

Alternatively, the importance of the sensitive samples can be increased by calculating sample-specific weights, which can be used as an additional input for the training of a weighted random forest. To this end, let $S = \{s_1, \dots , s_N\}$ be the set of cell lines, $y \in {\mathbb {R}}^N$ the response vector for a specific drug given as logarithmized IC50 values for the regression task. Let $Y \in \{0,1\}^N$ (0: resistant, 1: sensitive) be the binarized response vector derived from *y* by comparison to a drug-specific threshold *t*. We can use the ratio between the number of sensitive (minority) cell lines $N_{\text {Sens}}$ and the number of resistant cell lines $N_{\text {Res}}$ to set the initial sample-specific weight for each sample *i* to

1 $$\begin{aligned} w_{i}^* = {\left\{ \begin{array}{ll} 1, &{}\quad \text {if sample } i \text { is resistant} \\ \frac{N_{\text {Res}}}{N_{\text {Sens}}}, &{}\quad \text {if sample } i \text { is sensitive.}\\ \end{array}\right. } \end{aligned}$$

We call these weights *simple sample weights* (simple s.w.).

To emphasize the distance from the threshold *t*, we also investigated the usage of weight-functions that assign larger weights to samples that are further away from the binarization threshold as used in LOBICO^12^ (cf. Methods for a more detailed description).

#### Improving regression performance

To enhance the regression performance even more, we suggest alternative tree-weighting schemes. In particular, we propose to weight the trees as follows: If a sample is predicted to be sensitive, then we suggest considering only trees that agree with this class prediction (binary scheme by Rahman et al.). If a sample is predicted to be resistant, we suggest employing all trees and, hence, we use the usual RF weight, i.e., $\frac{1}{B}$. Here, *B* denotes the number of trees in the forest (cf. Methods for other tree weighting schemes). We refer to this tree-weighting scheme as *binary sensitive tree weighting* (binary sens. t.w.).

### Performance comparison of regression and classification RFs

We applied various versions of standard regression RFs, HARF, and our new approach SAURON-RF to 86 drugs of the GDSC. To evaluate the regression performance, we then calculated the mean squared error (MSE) and median squared error (median SE) averaged across all drugs. Additionally, we calculated the MSE and median SE for the set of sensitive and set of resistant cell lines separately to assess the influence of regression imbalance. To evaluate the classification performance of the regression forests, we determined sensitivity, specificity, and Matthew’s correlation coefficient (MCC). To derive class predictions for a standard regression RF (rRF), we compare its prediction to the drug-specific IC50 threshold.

Figure 3 shows an overview of the test results for 20 gene expression features obtained by our feature selection (additional results can be found in the Supplementary Material). As can be seen in Fig. 3A, both rRF and $\text {HARF}_{SR}$ have a low overall MSE, while they perform very poorly for the sensitive cell lines, i.e. the average MSE for the sensitive cell lines is 2.9 times (rRF) and 3.2 times ($\text {HARF}_{SR}$) as high as the average MSE of the resistant cell lines. Moreover, the statistical sensitivity for both approaches is very low with 10% for rRFs and 9% for $\text {HARF}_{SR}$. When counteracting class imbalance by employing simple sample weights as defined in Eq. (1), the sensitivity of both methods increases substantially by 21% and 50%, respectively. In comparison, the specificity decreases only moderately by 5% (rRF) and 15% ($\text {HARF}_{SR}$). Simultaneously, the average MSE for the sensitive cell lines drops from 3.99 to 2.68 (− 32.8%) for rRF and from 4.31 to 3.16 (− 26.6%) for $\text {HARF}_{SR}$. In contrast, the MSE for the resistant cell lines rises from 1.36 to 1.68 (23,5%) for rRF and from 1.34 to 1.74 (29.8%) for $\text {HARF}_{SR}$.

When combining the sample weights with the proposed tree weighting scheme (SAURON-RF, simple s.w., binary sens t.w.), the reduction in average MSE for the sensitive cell lines compared to rRF even amounts to 1.63 ($-40.8\%$), and compared to $\text {HARF}_{SR}$ amounts to 1.95 ($-45.2\%$). The MSE for the resistant cell lines increases by 0.55 (40.4%) for rRF and 0.57 (42.5%) for $\text {HARF}_{SR}$. In total, the MSE of the resistant and sensitive cell lines is more leveled for SAURON-RF, i.e. the average MSE for the sensitive cell lines is only 1.2 times as high as the average MSE of the resistant cell lines. We note that upsampling improves the predictive performance for the sensitive samples slightly less than the proposed simple sample weights. Hence, the overall best performance in terms of classification and regression was achieved by SAURON-RF simple s.w., binary sens. t.w.

For this best-performing model, we also plotted the absolute gain in performance for the sensitive cell lines against the absolute loss of performance for the resistant cell lines compared to rRF on a per drug basis (cf. Fig. 3B). For almost all drugs, the absolute performance gain of SAURON-RF for the sensitive cell lines outweighs the absolute performance loss for the resistant ones in terms of classification and regression. When we focus on the correctly identified sensitive cell lines, we note that the MSE value for SAURON-RF is even three times smaller than the MSE of all sensitive cell lines. Hence, our results also strongly indicate that further reducing the classification error would substantially improve regression performance.

To quantify whether our joint regression and classification approach can compete with a pure classification method, we compared the classification performance of SAURON-RF to classification RFs (cRFs) in terms of sensitivity, specificity, and MCC. To compare the regression performance, we also derived a continuous prediction for the cRF. For a new sample, we first determine the trees that agree with the majority vote of the forest. Then we average the continuous values of the training samples in the reached leaf nodes. Finally, we then average over the selected trees.

The cRF exhibits a similar classification performance to $\text {HARF}_{SR}$ and rRF: 99% specificity, 9% sensitivity, and an MCC of 0.12. When integrating our proposed sample weights into cRF (cRF, simple s.w.) or using the built-in class balancing of the cRF python implementation (cRF, balanced s.w.), the sensitivity and MCC improve. However, in terms of statistical sensitivity, SAURON-RF is superior, while still maintaining a similar specificity. Furthermore, SAURON-RF outperforms all tested cRF versions in the regression task.

Finally, we also compared SAURON-RF to a hierarchical RF approach, where we fit one classification RF on the complete data set and two regression RFs on the respective subsets of sensitive and resistant cell lines. The performance of this approach was also inferior to all versions of SAURON-RF presented in Fig. 3 (see Supplementary Fig. 17 for details).

To conclude, SAURON-RF substantially improves regression and classification performance for the sensitive cell lines at the cost of a moderate decline for the resistant ones.

### Evaluating predictive biomarkers for top-performing drug data sets

To assess the capability of our method to select cancer biomarkers that provide information on drug response, we investigated the importance of the input features in our drug-specific models. To this end, we only considered models trained using our best-performing version of SAURON-RF and selected the ten drugs with largest MCC. Then, we identified the most important features for each drug and investigated potential involvement in mechanisms of drug sensitivity or resistance through literature research. For most drugs (6/10), we could identify at least one feature with a known relation to drug response among the top five features (see Supplementary Tables 1–10 and Figs. 19, 20 for further details). For the BCL2 inhibitor ABT737, for example, the most important features include its target gene BCL2, as well as MIR22HG, IDH2 and BLVRB. While MIR22HG is involved in the downregulation of BCL2^31,32^, mutations in IDH2 are associated with increased sensitivity to ABT737^33,34^. Low expression of BLVRB has been linked to increased sensitivity to the BCL2 inhibitor Obatoclax^35^. For the drug Nutlin-3a(-), which targets the p53 pathway, we identified its direct target gene MDM2 as most important feature. Additionally, we identified RPS27L – a downstream target of TP53 – that is activated in cells, which undergo apoptosis following Nutlin-3a(-) treatment^36^. Further important features included DDB2 and CYFIP2, whose expression has been shown to increase through Nutlin-3a(-) treatment^37,38^. This could indicate the involvement of these genes in the mode of action of Nutlin-3a(-).

We also investigated whether we are able to identify features that are more commonly associated with drug sensitivity or resistance. These results are presented in the Supplementary Material (Supplementary Figs. 21–30).

## Discussion

The primary goal of personalized medicine in cancer is to optimize (drug) treatment for given tumors. To study the relationship between drug sensitivity and cellular characteristics, ML methods are frequently used. However, the skewed distribution of drug response values in favor of the more drug-resistant cell lines negatively affects both the classification and regression performance for the sensitive cell lines.

To address these issues, we developed SAURON-RF, an RF-based method that simultaneously performs classification and regression. We could not only substantially improve the classification performance for the sensitive cell lines but also the respective regression performance at the expense of a moderate loss in performance for the resistant cell lines. Furthermore, our results indicate that an accurate classification can substantially improve a subsequent regression. We show that the practical implementation of this strategy could be best achieved using a combined regression and classification approach. Therefore, the development and refinement of joint classification and regression approaches like SAURON-RF seem to be a promising field of future research.

A prediction tool like SAURON-RF can be integrated into the medical decision process as part of a comprehensive decision support pipeline. We plan to incorporate SAURON-RF into our decision support pipeline $\text {ClinOmicsTrail}^{bc}$^39^, which helps to interpret molecular and clinical data of patients and, amongst others, assesses standard-of-care treatments, the pharmacokinetics of drugs, potential adverse effects, as well as known biomarkers for drug efficacy. Our tool could not only provide evidence for on-label use of drugs but also give additional insight into potential off-label treatment options.

However, we recognize several potential starting points for improving our method especially concerning statistical performance and interpretability.

Since we have shown that joint regression and classification is advantageous for both classification and regression compared to each of them alone, a potential extension to SAURON-RF could be to combine regression and classification splitting criteria in the tree-building process, e.g., as described in^40,41^.

Another important factor that influences the predictive performance of our method is the choice of input features. A current drawback of our method is that we solely focus on gene expression data. While it has been shown that gene expression is the most informative data type^5^, the performance and interpretability of the machine learning models can for example benefit from the integration of (epi)genomics data^5^, protein-protein interaction networks^14^, pathway information^4^, and pharmacogenomic a priori knowledge^13^.

An additional factor that influences the predictive performance of our approach is the class division into sensitive and resistant cell lines. Future versions of our method could include a more fine-grained division, e.g. four classes (highly sensitive, sensitive, resistant, highly resistant).

Currently, we only use SAURON-RF to perform monotherapy prediction. However, targeted monotherapies often promote the development of drug resistances. Since combination therapies usually improve therapeutic response and help overcome drug resistance^42^, the prediction of synergistic drug combinations, i.e., drug synergy, would be a desirable next step. Here we could leverage information from databases such as DrugComb^42^ or combine our approach with drug synergy prediction methods such as DeepSynergy by Preuer et al.^19^ or REFLECT by Li et al.^20^. Such approaches usually employ chemical fingerprints of drugs and exploit drug similarities to perform drug combination predictions. Currently, SAURON-RF does not consider drug similarities since we train single-target (drug) models. However, the incorporation of drug-based features can also be beneficial for monotherapy prediction^9,15–18^.

To conclude, the presented analyses demonstrate that SAURON-RF can effectively counteract classification and regression imbalance by simultaneously performing classification and regression in an RF model. In particular, we show that SAURON-RF improves predictions for the underrepresented class of drug-sensitive samples, which is a further step towards the goal of personalized medicine in cancer.

## Methods

### Data acquisition

For all our analyses, we employ data from the Genomics of Drug Sensitivity in Cancer (GDSC) cancer cell line panel Release 8.3 (June 2020). In particular, we downloaded the pre-processed gene expression data (Affymetrix Human Genome U219 Array) and drug sensitivity data (GDSC1 compounds: Syto60 and resazurin assay, GDSC2 compounds: CellTiter-Glo assay) in the form of IC50 values.

### Processing of drug response data

In our analyses, we focus on the more recently published GDSC2 data set, which is based on an improved drug screening procedure and assay compared to GDSC1. In total, the GDSC2 data set consists of 809 cell lines and 198 drugs. We use only drugs with more than 750 available cell lines, which results in 86 investigated drugs. For each drug, we consider only cell lines with complete information for drug sensitivity and gene expression.

Since SAURON-RF performs a simultaneous regression and classification analysis, it requires a continuous and a binary response vector. For the regression response of our method, we use the provided logarithmized IC50 values. For the classification task, we derive a discretized (binary) drug response from the continuous IC50 values. To this end, we employ the procedure introduced by Knijnenburg et al.^12^ and apply it using a custom R-script as described previously^13^. For each drug, this procedure results in an IC50 threshold that can then be employed to decide for each cell line whether it is sensitive or resistant to a particular drug, which results in a binary drug-specific response vector.

### Model training

We employ the following joint model training and evaluation scheme for all of the methods used in this paper: We derive a training (80%) and test data set (20%) for each drug by randomly drawing without replacement from the set of available cell lines. We perform a 5-fold CV on the training set, which we use to assess model quality. We then train the final models on the complete training data set and apply the fitted models to the test set to derive a test error.

Since the gene expression data is very high-dimensional, we implemented a feature selection that reduced the number of features before serving as input for the machine learning methods, i.e., the feature selection is performed before the training of each model and also during the CV. The optimal number of input features is a hyperparameter, which can be determined during the CV (see Supplementary Material). In particular, we tested $P_{opt} \in \{20,40,60,80,100\}$. The used feature selection is based on the minimum-redundancy-maximum relevance principle. More specifically, we implemented the greedy minimum-redundancy-maximum-relevance algorithm proposed in^43^. The algorithm tries to maximize the feature-response (gene-drug) mutual information (MI) while minimizing the feature-feature (gene-gene) MI. In the Supplementary Material, we provide further information on the used parameters for the feature selection.

### Implementation

The implementation of all random forest-based approaches, including the HARF approach as well as SAURON-RF, was accomplished using python 3.0 and the sklearn RandomForestRegressor and RandomForestClassifier packages^44^. Boosting trees and elastic nets were fit using the R packages gbm (Version 2.1.8) and glmnet (Version 4.1.1), respectively. The neural networks were fit using the python framework Keras (Version 2.3.1) and together with the GPU Version of Tensorflow (1.13.1). We provide a table with the used (hyper)parameters for all methods in the Supplementary Material.

### Machine learning methods for drug sensitivity prediction

The goal of our novel approach called SAURON-RF (SimultAneoUs Regression and classificatiON Random Forest) is to tackle the drug sensitivity prediction problem by using random forests simultaneously for regression and classification. To this end, we pursue a similar strategy to HARF^24^, i.e., we train regression random forests and augment them with class information. However, instead of using cancer types as classes, we use the presented drug-specific division into sensitive and resistant cell lines. For most drugs, the number of sensitive cell lines is much lower than the number of resistant cell lines. We account for this class imbalance by upsampling or sample-specific weights. Moreover, we propose other tree-weighting schemes. We first discuss regression random forests and HARF before we describe our novel approach SAURON-RF in detail.

#### Random forests

Random forests are a tree-based ensemble approach that can be used for classification and regression^45^. In the following description, we focus on random forest regression since this is the basis of SAURON-RF.

Let $S = \{s_1, \dots , s_N\}$ be the set of samples (e.g., cell lines) and $F = \{f_1, \dots , f_P\}$ be the set of features (e.g., gene expression values). Suppose $X \in {\mathbb {R}}^{N \times P}$ is the model matrix and $y \in {\mathbb {R}}^N$ the response vector (e.g., logarithmized IC50 values) for the regression task. Let *B* be the number of trees in the forest. Each tree $b \in \{1, \dots , B\}$ of the forest is built on a bootstrapped training sample, i.e., for each tree, *N* samples are drawn with replacement from the set of samples. A single decision tree is then built as described below. Starting with a root node containing all bootstrap samples, the following steps are repeated until some stopping criterion such as the minimal number of samples per leaf is fulfilled:

- For each current leaf node that does not yet fulfil the stopping criterion, draw $m < P$ features without replacement from the set of features.
- For each drawn feature, assess the quality of its putative splitting points, i.e., determine by how much a binary division of the samples at each specific splitting point can improve the used error measure.
  To this end, let *v* be the current node and let $\delta (v)$ represent all bootstrap samples that belong to this node. Let $y^v$ be the known response vector for the subset of bootstrap samples falling into node *v* and let $w_n^v$ be sample weights that reflect the desired importance of each sample. These sample weights then influence the quality assessment of the split in the error measure. Typically, all samples are weighted equally. However, the weights can be set to meet user-defined properties, which we will discuss in-depth when we introduce SAURON-RF.
  For regression, measures such as the mean squared error (MSE) between the known response $y^v$ and the predicted response ${\hat{y}}^v$ of a node *v* can be used to assess the quality of the split. The predicted response for each sample *i* of a node is the (weighted) average of the response values of the samples in the node and can be calculated as follows:
  2 $$\begin{aligned} \forall i \in \delta (v) :{\hat{y}}_{i}^v = \sum _{n \in \delta (v)} w_n^{v} \cdot y_n^v.\end{aligned}$$
  The sample-specific weight $w_n^{v}$ is set to $\frac{1}{\vert \delta (v)\vert }$ if an ordinary average is desired.
  The (weighted) MSE measures the deviance of the known response values from the predicted ones and is defined as
  3 $$\begin{aligned} MSE(y^v, {\hat{y}}^v) = \sum _{n \in \delta (v)} w_n^{v} \cdot (y_n^v - {\hat{y}}_n^v)^2.\end{aligned}$$
  Similarly, the sample-specific weight $w_n^{v}$ can be set to $\frac{1}{\vert \delta (v)\vert }$ to calculate the ordinary MSE. The improvement of the error by splitting node *v* into the two nodes $v_r$ and $v_l$ can consequently be calculated as
  4 $$\begin{aligned}&w_{an}(v) \cdot ( MSE(y^v, {\hat{y}}^v) \\&\quad -w_{ch}(v_r) \cdot MSE(y^{v_r}, {\hat{y}}^{v_r}) \nonumber \\&\quad - w_{ch}(v_l) \cdot MSE(y^{v_l}, {\hat{y}}^{v_l})).\nonumber \end{aligned}$$
  Here, $w_{an}(v)$, $w_{ch}(v_r)$, and $w_{ch}(v_l)$ are node-specific weights for the ancestor and child nodes respectively, which can for example represent the fraction of samples assigned to a node. In particular, $w_{an}(v) = \frac{\vert \delta (v)\vert }{N}$, $w_{ch}(v_r) = \frac{\vert \delta (v_r)\vert }{\vert \delta (v)\vert }$, and $w_{ch}(v_l) = \frac{\vert \delta (v_l)\vert }{\vert \delta (v)\vert }$ are typical choices for a regression forest^44^.
- Use the feature and splitting point for which Eq. (4) is minimized to divide the samples into two groups. The respective splitting criterion of the feature then represents an internal node of the tree, and the two groups become the children of this node.

For a new sample $x' \in {\mathbb {R}}^P$, the prediction of a single tree *b* can then be calculated as the average of the response values in the reached leaf node. Let $\mu$ be the leaf node (partition) that is reached by sample $x'$, then the prediction of a single tree can be calculated as described above (cf. Eq. 2)

5 $$\begin{aligned} {\hat{f}}_b (x') = \sum _{ n \in \delta (\mu )} w_{n}^{\mu } \cdot y_n^{\mu }. \end{aligned}$$

The prediction of the random forest can subsequently be obtained by averaging the predictions of all of the trees. Similar to the sample-specific weights, the trees can be assigned weights that quantify their importance for the prediction and the formula for the prediction is given by

6 $$\begin{aligned} {\hat{f}}(x') = \sum _{b = 1}^{B} w_b(x') \cdot {\hat{f}}_b(x'). \end{aligned}$$

Here, $w_b(x')$ is the tree-specific weight, which is set to $\frac{1}{B}$ in a conventional random forest to obtain the simple average of the trees. In the following sections, we discuss other options for tree weighting.

#### Heterogeneity-aware random forests (HARF)

The HARF method builds the forest as described in the previous section. However, it uses additional class information of the samples to modify the weight of the trees for the final prediction (Eq. 6). Let $C = \{c_1, \dots , c_K\}$ be a set of different classes (e.g. different cancer types) that can be assigned to the samples. Rahman et al. assign each leaf node to the class that represents the relative majority (mode) of the classes of all training samples in that leaf node. This means that each leaf node does not only make a prediction for the continuous response but also for the class of a sample. For a new sample $x'$, the prediction of a tree is then given by the prediction of the leaf that is reached by this sample. The predicted class of the complete forest for $x'$ is defined as the mode over the class predictions of all trees. Based on this classification, the prediction of the regression value is then accomplished using only the trees for which the predicted class is equal to the class prediction of the forest . Accordingly, the tree-specific weights $w_{b}(x')$ are calculated by dividing an indicator variable $I_b(x')$, which is 1 if the mode of the leaf of tree *b* is equal to the mode of the forest and 0 otherwise, by the sum of these indicator variables over all reached leaves in all trees, i.e., the weight $w_{b}(x')$ becomes

7 $$\begin{aligned} w_{b} (x') = \frac{I_b(x')}{\sum _{\beta =1}^{B} I_{\beta }(x')}.\end{aligned}$$

We refer to this tree weighting as *binary tree weighting* scheme (binary t.w.).

#### Simultaneous regression and classification random forests (SAURON-RF)

We propose several modifications to HARF that can improve both classification and regression performance. In Fig. 1, we give an overview of the three main modifications that can be summarized as follows: While Rahman et al. integrate heterogeneity in terms of different cancer types as classes into their regression, we suggest using the division into sensitive and resistant cell lines that is derived from the continuous response values. We then counteract the observed class imbalance between sensitive and resistant samples with either upsampling or sample-specific weights. Finally, we suggest using a different tree weighting scheme to improve the performance of the RF.

SAURON-RF employs the explained division of sensitive and resistant cell lines to simultaneously perform classification in a random forest regression model. Thus, the following description of our method will be limited to the case with two classes $C = \{0,1\}$ (e.g., 1: sensitive, 0: resistant). However, the method can be extended to multiple classes.

One possibility to counteract class imbalance is the use of sample-specific weights, which directly influence the training process through Eqs. (2)–(5). We suggest several possibilities to set the sample weights. A simple way to set the weights is to utilize the proportion between the majority and minority. To this end, let $Y \in \{0,1\}^{N}$ be the binarized response vector derived from discretizing the continuous response vector *y* by comparison to a threshold *t*. W.l.o.g., let 0 be the majority and 1 be the minority class. Let $N_{Sens}$ be the number of sensitive samples and $N_{res}$ be the number of resistant samples. The initial weight for each sample can be set to

8 $$\begin{aligned} w_{i}^* = {\left\{ \begin{array}{ll} 1, &{} \text {if sample } i \text { is resistant} \\ \frac{N_{\text {Res}}}{N_{\text {Sens}}}, &{} \text {if sample } i \text { is sensitive.}\\ \end{array}\right. } \end{aligned}$$

We refer to these weights as *simple sample weights* (simple s.w.). Alternatively, we propose to use weights that emphasize samples with sensitivity values that have a higher distance from the threshold *t*. This approach was introduced for the LOBICO method^12^ and slightly modified in MERIDA^13^. We then calculate the initial weights for all training samples as

9 $$\begin{aligned} w_i^{*} = \frac{\vert y_i - t\vert ^d}{2 \cdot \sum _{\forall n \in \{1, \dots , N\}: Y_n = Y_i} \vert y_n - t\vert ^d} \end{aligned}$$

with $d \in \{1,2\}$. Here, the initial weight corresponds to the distance of the sensitivity value from the threshold normalized such that the total weights for one class equal 0.5. We name the corresponding weights according to the exponent, i.e., *linear* or *quadratic*.

Once the initial weights have been determined, these weights are carried through the training procedure of the random forest. More precisely, the sample-specific weights for each node *v* can be calculated as follows

10 $$\begin{aligned} w_{i}^{v} = \frac{w_i^{*}}{\sum _{n \in \delta (v)} w_{n}^{*}} \end{aligned}$$

and the node-specific weights in Eq. (4) become

11 $$\begin{aligned} w_{an}(v) = \frac{\sum _{\forall n \in \delta (v)} w_{n}^{*}}{\sum _{\forall n \in \delta (root(v))} w_{n}^{*}} \end{aligned}$$

and

12 $$\begin{aligned} w_{ch}(v) = \frac{\sum _{\forall n \in \delta (v)} w_{n}^{*}}{\sum _{\forall n \in \delta (parent(v))} w_{n}^{*}}. \end{aligned}$$

Here, *root*(*v*) is the root of tree *b* and *parent*(*v*) is the direct ancestor of *v* in this tree.

Alternatively to calculating sample-specific weights to assign samples a higher importance during training, we propose to perform upsampling prior to the training of the forest. In particular, we suggest upsampling the minority class by random drawing with replacement from the set of sensitive samples until the number of samples from the minority and majority class is equal. We call this method *upsampling*. We also investigated an upsampling scheme, which we call *proportional upsampling*, where we consider the distance from the threshold *t* to determine how often a sensitive sample should be duplicated. To this end, we calculate the linear sample-specific weights (see Eq. 9) for the sensitive cell lines. Then we multiply these weights by 2 such that they sum up to 1. For each sample, this value then reflects the percentage of importance. We then determine the desired number of copies by multiplying this importance with the total number of resistant samples.

Finally, we suggest alternative tree weighting schemes to the binary one by Rahman et al. to improve the performance of our method. To this end, we attach the class assignments to the training samples in the leaf nodes and determine the mode of class assignments per leaf as described above. Thus, each tree can predict a class depending on the reached leaf, and the majority vote over all tree predictions is considered the class prediction. Based on this prediction, the trees obtain weights.

For the tree weighting scheme referred to as *binary sensitive (binary sens.)*, we use the HARF weights (c.f. Eq. 7) for the samples predicted to be sensitive, while we use the usual random forest prediction, i.e., tree weight $\frac{1}{B}$, for the samples predicted to be resistant. For the tree weighting scheme called *majority tree weighting*, we determine for each leaf the weighted fraction of samples that agree with the class prediction of the forest. For a particular tree, we use this fraction in the reached leaf normalized by the sum over all fractions as tree weight. Let $I_n(x')$ be an indicator function that is 1 if the class of sample *n* is equal to the overall majority of trees and 0 if not. Let $\mu$ be the reached leaf node of tree *b*. With the fraction given by

13 $$\begin{aligned} \text {frac}_{b} (x') = \frac{\sum _{\forall n \in \delta (\mu )} I_n(x') \cdot w_n^{*}}{ \sum _{\forall n \in \delta (\mu )} w_n^{*}} \end{aligned}$$

the tree-specific weight is given as

14 $$\begin{aligned} w_{b} (x') = \frac{\text {frac}_{b}}{ \sum _{\beta =1}^{B} \text {frac}_{\beta }(x')}. \end{aligned}$$

Finally, we also used a tree weighting scheme, which we call *majority sensitive (majority sens.)*, where we employ this weight for the sensitive samples and the usual RF weight $\frac{1}{B}$ for the resistant samples.

## Supplementary Information

Supplementary Information.

## Data Availability

The used drug sensitivity data sets (gene expression and drug response data from Release 8.3, June 2020) were downloaded from the publicly available repository of the GDSC database (https://www.cancerrxgene.org/downloads/bulk_download). All other material is made available in the Supplementary Material or via the github page for this manuscript (https://github.com/unisb-bioinf/SAURON-RF.git).

## Code availability

The corresponding code is available on github (https://github.com/unisb-bioinf/SAURON-RF.git).
