# Supplementary material for: Simultaneous regression and classification for drug sensitivity prediction using an advanced random forest method
Source: Sci Rep. 2022 Aug 5;12:13458. doi: 10.1038/s41598-022-17609-x (PMC9356072; doi:10.1038/s41598-022-17609-x)
Supplement: Supplementary file 1 — Supplementary Information. [file 41598_2022_17609_MOESM1_ESM.pdf]

# - Supplement -
Simultaneous regression and classification for drug sensitivity prediction using an advanced random forest method

Kerstin Lenhof, Lea Eckhart, Nico Gerstner, Tim Kehl, and Hans-Peter Lenhof

1 Feature Selection

Since the gene expression data provided in the GDSC database is high-dimensional, we applied a heuristic feature selection by Kwak et al. (2002) to reduce the number of input features for our models. Their approach is based on the minimum-redundancy-maximum-relevance principle, which aims to select features that have a strong dependence on the response variable(s) (i.e., large relevancy) but weak dependence on each other (i.e., small redundancy). To measure dependence between two variables, Kwak et. al employ the mutual information.
Let F denote the set of all potential input features and C the response variable. Furthermore, let K be the number of features to be selected and S the set of already selected features, which is initially empty. Now, we iteratively add that feature $f_i \in F$ to S, which maximizes the following term:

$$\max_{f_i \in F} I(C; f_i) - \sum_{f_s \in S} \frac{I(C; f_s)}{H(f_s)} \cdot I(f_i; f_s) \quad (1)$$

Here, $I(a; b)$ is the mutual information between a and b and $H(a)$ is the entropy of a. After a feature f_i is selected, F and S are updated:

$$F \leftarrow F \backslash \{f_i\}, \quad S \leftarrow S \cup \{f_i\} \quad (2)$$

This procedure is repeated until the desired number of features is selected, i.e., until $|S| = K$.
For each drug, we employ this approach to generate drug-specific feature sets that serve as input for all our models. In our application case, the features are the gene expression values and the output variable is the sensitivity of a particular drug given as logarithmized IC50. Since both gene expression and IC50 values are continuous, they must be

discretized to calculate their mutual information. To this end, we applied an equal width binning to partition the samples of each feature and each drug response variable into six bins. Instead of setting F to the full set of available gene expression features, we set F to the set of 1000 genes for which the mutual information to the investigated drug was largest, and analyzed different feature set sizes $K \in \{20, 40, 60, 80, 100\}$. As the applied feature selection is a greedy approach, where features are added iteratively, all features contained in smaller feature sets are also contained in the larger ones.

2 Additional Performance Results

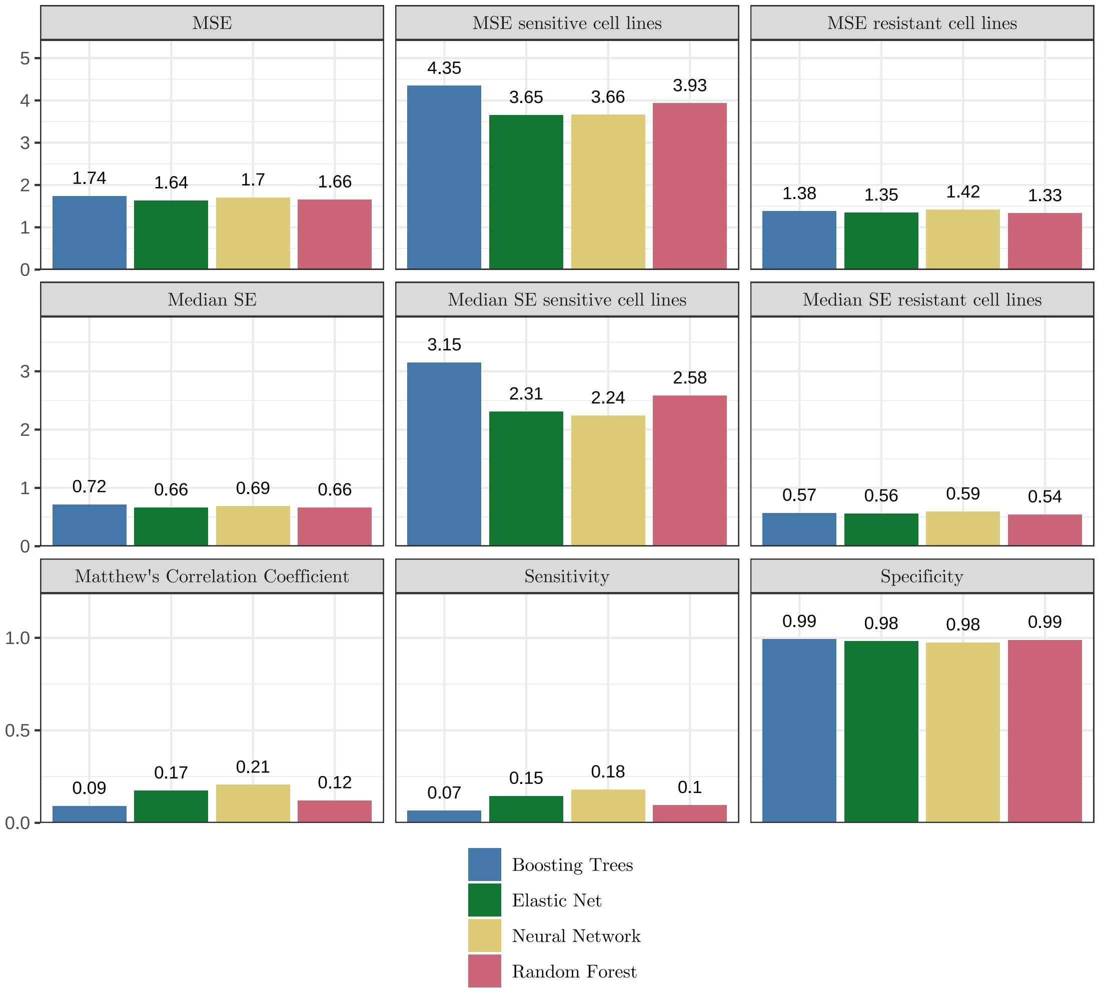

Figure 1: Test set performance for different machine learning algorithms. In this figure, we compare boosting trees, elastic nets, neural networks, and random forests. We show the average test set performance across the 86 different drugs for 20 input features.

ABT737

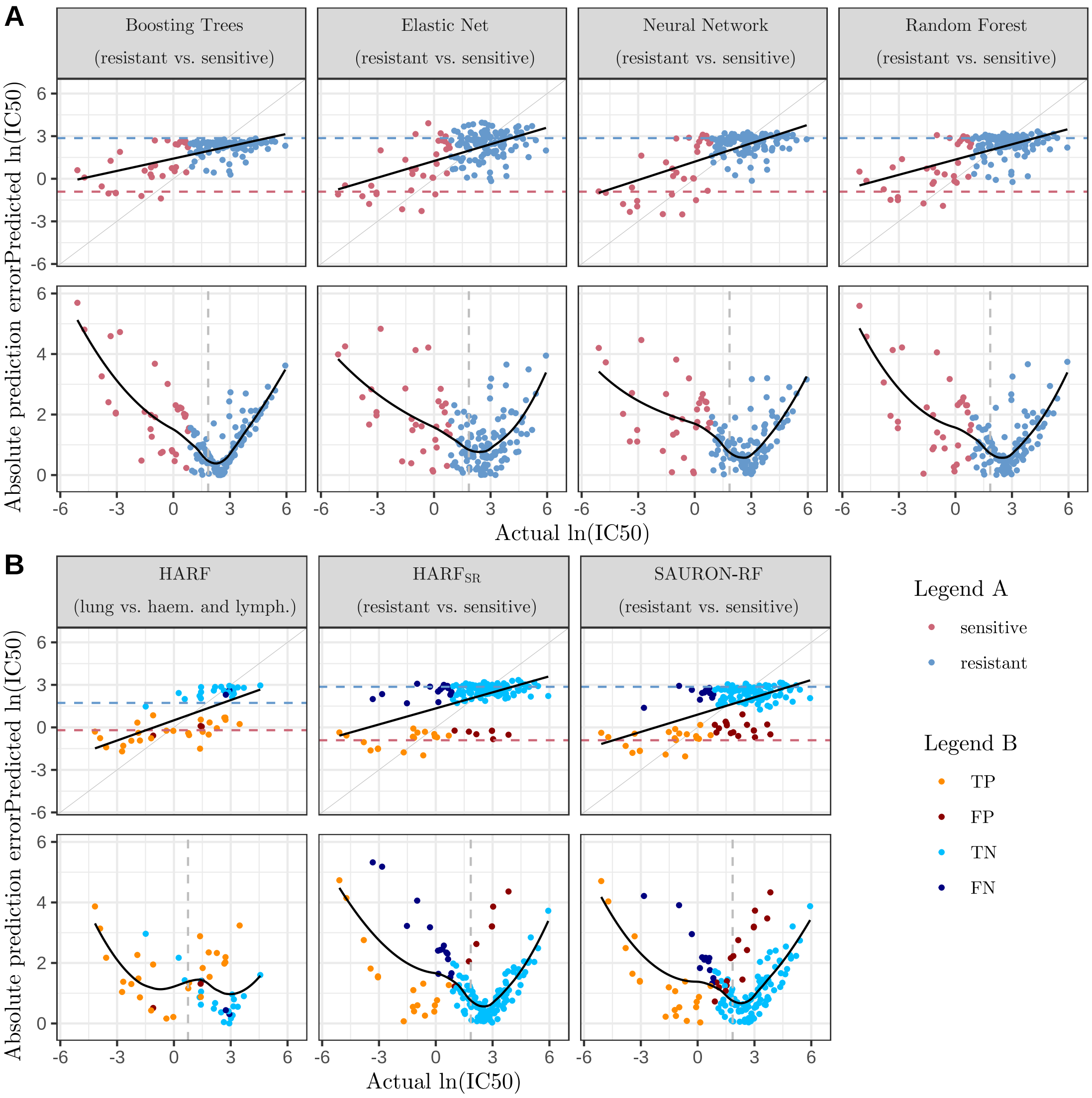

Figure 2: Regression performance of different ML methods for ABT737. This figure exemplifies the performance of different ML algorithms when applied to the ABT737 data set of the GDSC database using 20 input features. The upper rows of Fig. A and B show the predicted IC50 values plotted against the actual IC50 values including a fitted regression line, which is shown as a solid black line. The mean IC50 of training samples for each investigated class is depicted as a horizontal dashed line. The lower rows show the absolute prediction error. Here, the solid curve is a loess curve fitted to the error, the vertical dashed line gives the mean IC50 of all training samples. In Fig. A, we compare boosting trees, elastic net, neural networks and random forests. The point colouring indicates the class assignment (sensitive or resistant). The first plot in Fig. B depicts the performance of the original HARF algorithm applied to a restricted version of this data set containing only cell lines from two cancer types with different average drug responses, i.e., haematopoietic/lymphoid cell lines and lung cell lines. The second plot shows the performance of HARF when applied to our proposed class division (HARF$_{SR}$), and the last plot depicts the performance of the suggested SAURON-RF algorithm (SAURON-RF simple s.w., binary sens t.w.). Here, the point colouring represents the classification performance, i.e. we depict true positives (TP), false positives (FP), true negatives (TN), and false negatives (FN).

Afatinib

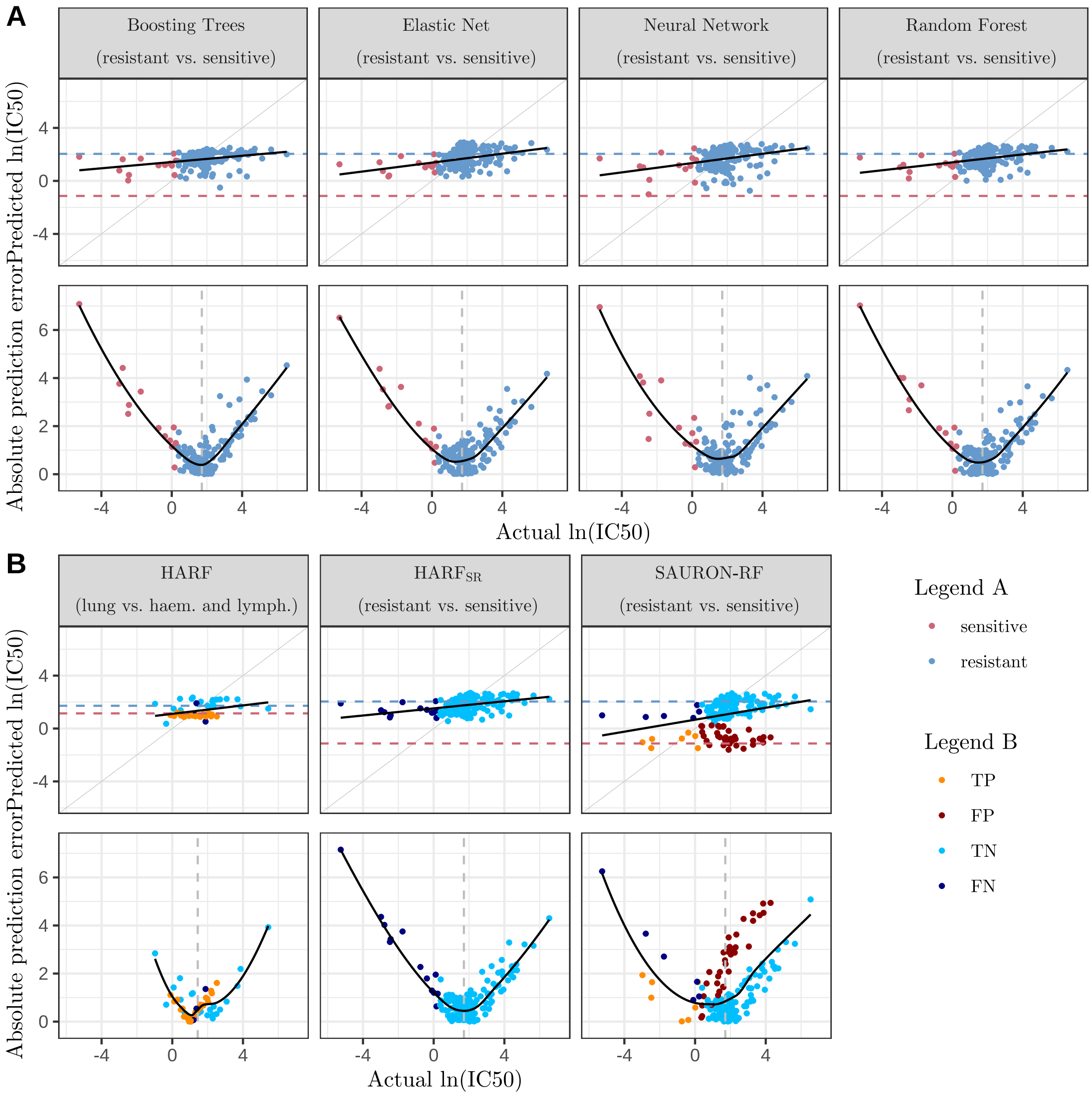

Figure 3: Regression performance of different ML methods for Afatinib. This figure exemplifies the performance of different ML algorithms when applied to the Afatinib data set of the GDSC database using 20 input features. The upper rows of Fig. A and B show the predicted IC50 values plotted against the actual IC50 values including a fitted regression line, which is shown as a solid black line. The mean IC50 of training samples for each investigated class is depicted as a horizontal dashed line. The lower rows show the absolute prediction error. Here, the solid curve is a loess curve fitted to the error, the vertical dashed line gives the mean IC50 of all training samples. In Fig. A, we compare boosting trees, elastic net, neural networks and random forests. The point colouring indicates the class assignment (sensitive or resistant). The first plot in Fig. B depicts the performance of the original HARF algorithm applied to a restricted version of this data set containing only cell lines from two cancer types with different average drug responses, i.e., haematopoietic/lymphoid cell lines and lung cell lines. The second plot shows the performance of HARF when applied to our proposed class division (HARF$_{SR}$), and the last plot depicts the performance of the suggested SAURON-RF algorithm (SAURON-RF simple s.w., binary sens t.w.). Here, the point colouring represents the classification performance, i.e. we depict true positives (TP), false positives (FP), true negatives (TN), and false negatives (FN).

Afuresertib

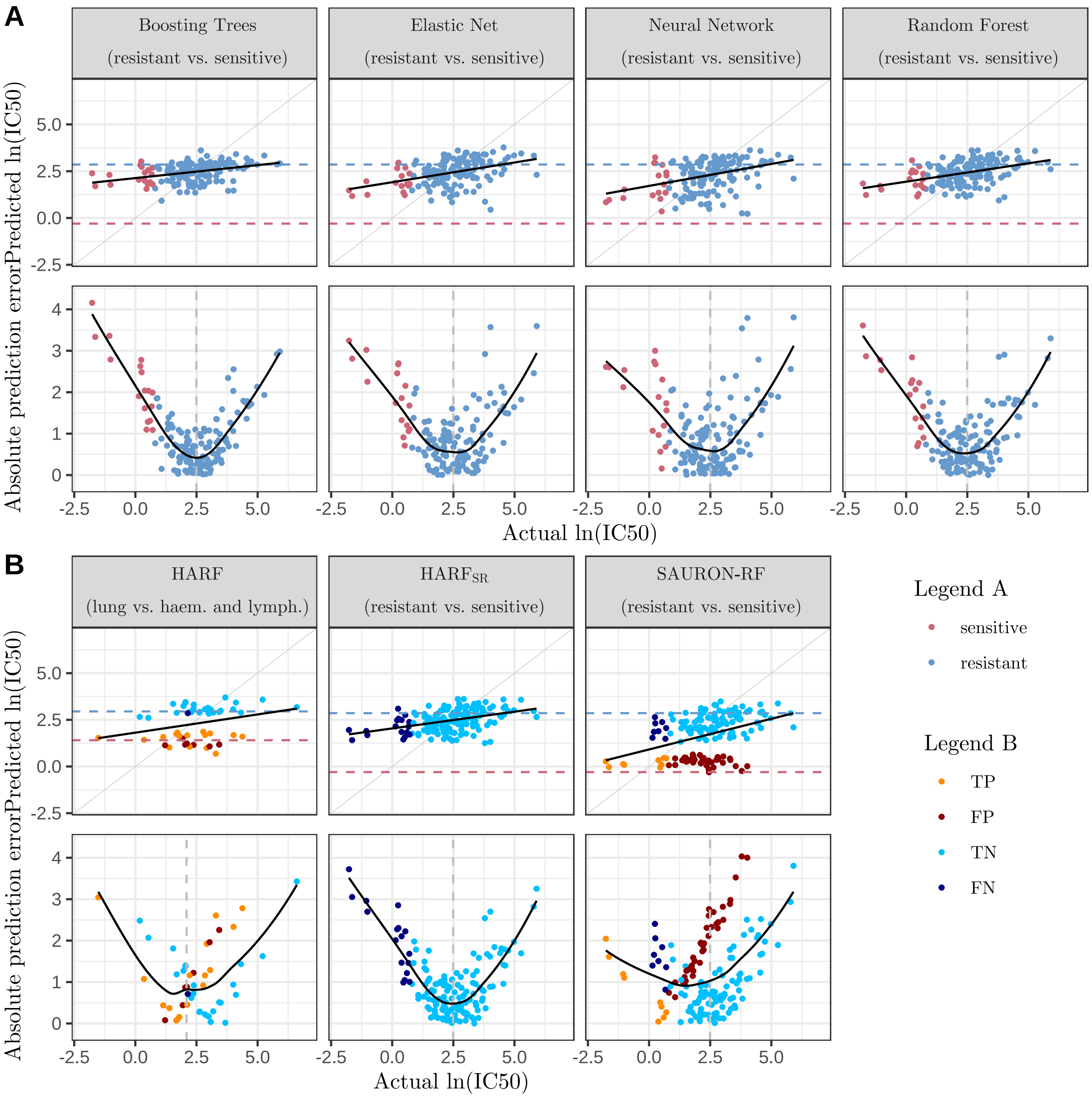

Figure 4: Regression performance of different ML methods for Afuresertib. This figure exemplifies the performance of different ML algorithms when applied to the Afuresertib data set of the GDSC database using 20 input features. The upper rows of Fig. A and B show the predicted IC50 values plotted against the actual IC50 values including a fitted regression line, which is shown as a solid black line. The mean IC50 of training samples for each investigated class is depicted as a horizontal dashed line. The lower rows show the absolute prediction error. Here, the solid curve is a loess curve fitted to the error, the vertical dashed line gives the mean IC50 of all training samples. In Fig. A, we compare boosting trees, elastic net, neural networks and random forests. The point colouring indicates the class assignment (sensitive or resistant). The first plot in Fig. B depicts the performance of the original HARF algorithm applied to a restricted version of this data set containing only cell lines from two cancer types with different average drug responses, i.e., haematopoietic/lymphoid cell lines and lung cell lines. The second plot shows the performance of HARF when applied to our proposed class division (HARF$_{SR}$), and the last plot depicts the performance of the suggested SAURON-RF algorithm (SAURON-RF simple s.w., binary sens t.w.). Here, the point colouring represents the classification performance, i.e. we depict true positives (TP), false positives (FP), true negatives (TN), and false negatives (FN).

AGI-5198

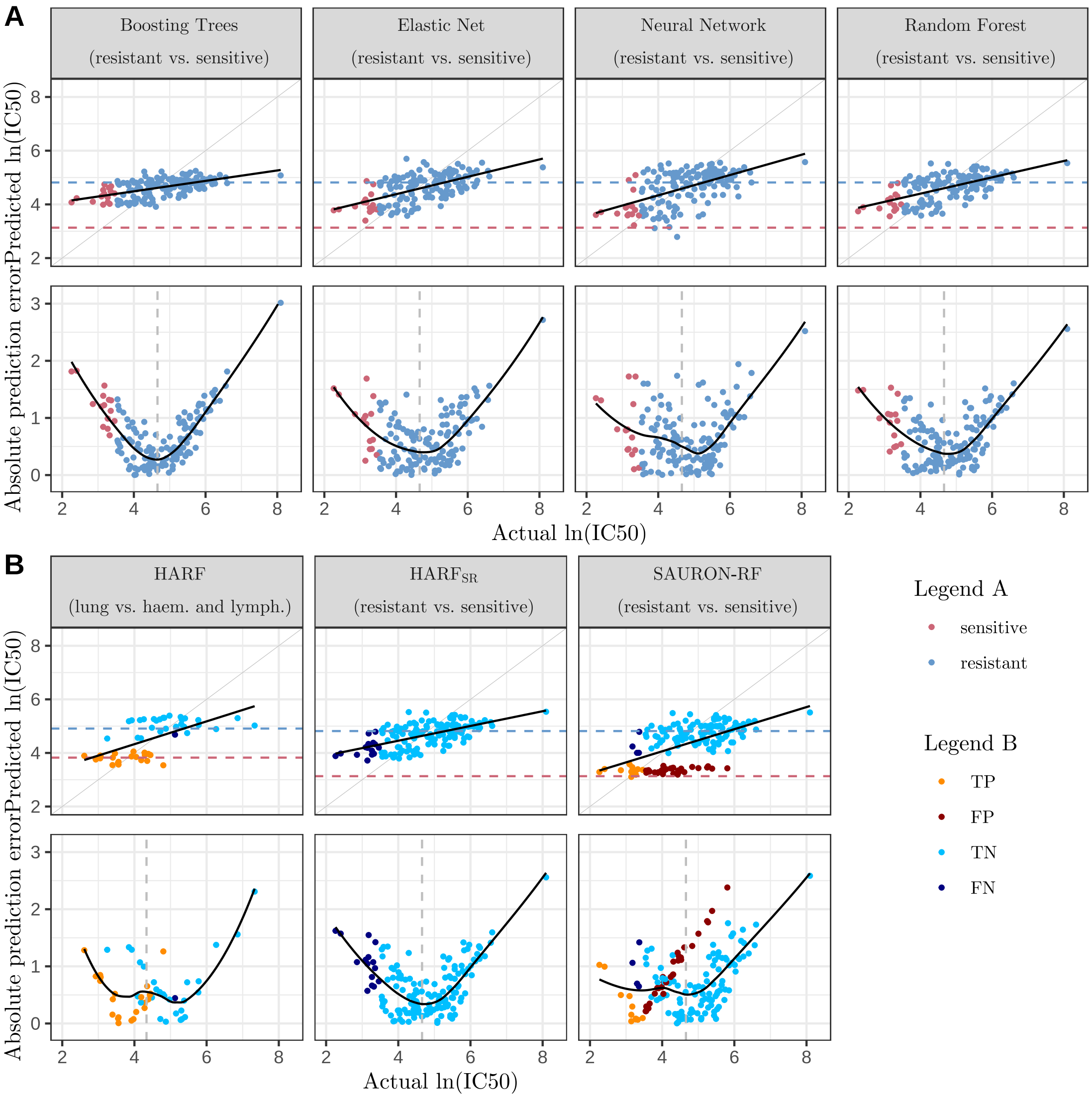

Figure 5: Regression performance of different ML methods for AGI-5198. This figure exemplifies the performance of different ML algorithms when applied to the AGI-5198 data set of the GDSC database using 20 input features. The upper rows of Fig. A and B show the predicted IC50 values plotted against the actual IC50 values including a fitted regression line, which is shown as a solid black line. The mean IC50 of training samples for each investigated class is depicted as a horizontal dashed line. The lower rows show the absolute prediction error. Here, the solid curve is a loess curve fitted to the error, the vertical dashed line gives the mean IC50 of all training samples. In Fig. A, we compare boosting trees, elastic net, neural networks and random forests. The point colouring indicates the class assignment (sensitive or resistant). The first plot in Fig. B depicts the performance of the original HARF algorithm applied to a restricted version of this data set containing only cell lines from two cancer types with different average drug responses, i.e., haematopoietic/lymphoid cell lines and lung cell lines. The second plot shows the performance of HARF when applied to our proposed class division (HARF$_{SR}$), and the last plot depicts the performance of the suggested SAURON-RF algorithm (SAURON-RF simple s.w., binary sens t.w.). Here, the point colouring represents the classification performance, i.e. we depict true positives (TP), false positives (FP), true negatives (TN), and false negatives (FN).

Alpelisib

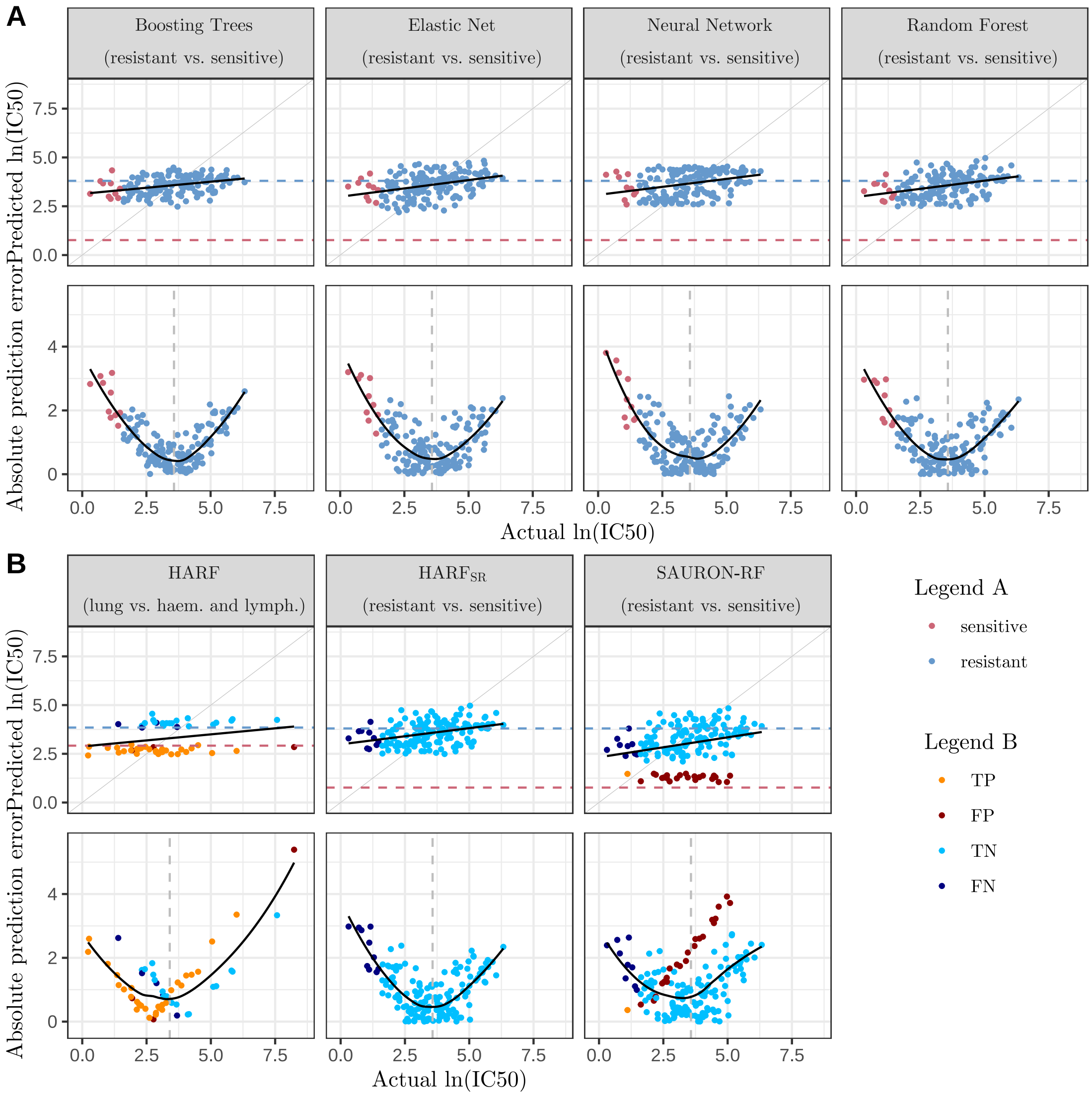

Figure 6: Regression performance of different ML methods for Alpelisib. This figure exemplifies the performance of different ML algorithms when applied to the Alpelisib data set of the GDSC database using 20 input features. The upper rows of Fig. A and B show the predicted IC50 values plotted against the actual IC50 values including a fitted regression line, which is shown as a solid black line. The mean IC50 of training samples for each investigated class is depicted as a horizontal dashed line. The lower rows show the absolute prediction error. Here, the solid curve is a loess curve fitted to the error, the vertical dashed line gives the mean IC50 of all training samples. In Fig. A, we compare boosting trees, elastic net, neural networks and random forests. The point colouring indicates the class assignment (sensitive or resistant). The first plot in Fig. B depicts the performance of the original HARF algorithm applied to a restricted version of this data set containing only cell lines from two cancer types with different average drug responses, i.e., haematopoietic/lymphoid cell lines and lung cell lines. The second plot shows the performance of HARF when applied to our proposed class division (HARF$_{SR}$), and the last plot depicts the performance of the suggested SAURON-RF algorithm (SAURON-RF simple s.w., binary sens t.w.). Here, the point colouring represents the classification performance, i.e. we depict true positives (TP), false positives (FP), true negatives (TN), and false negatives (FN).

AZD3759

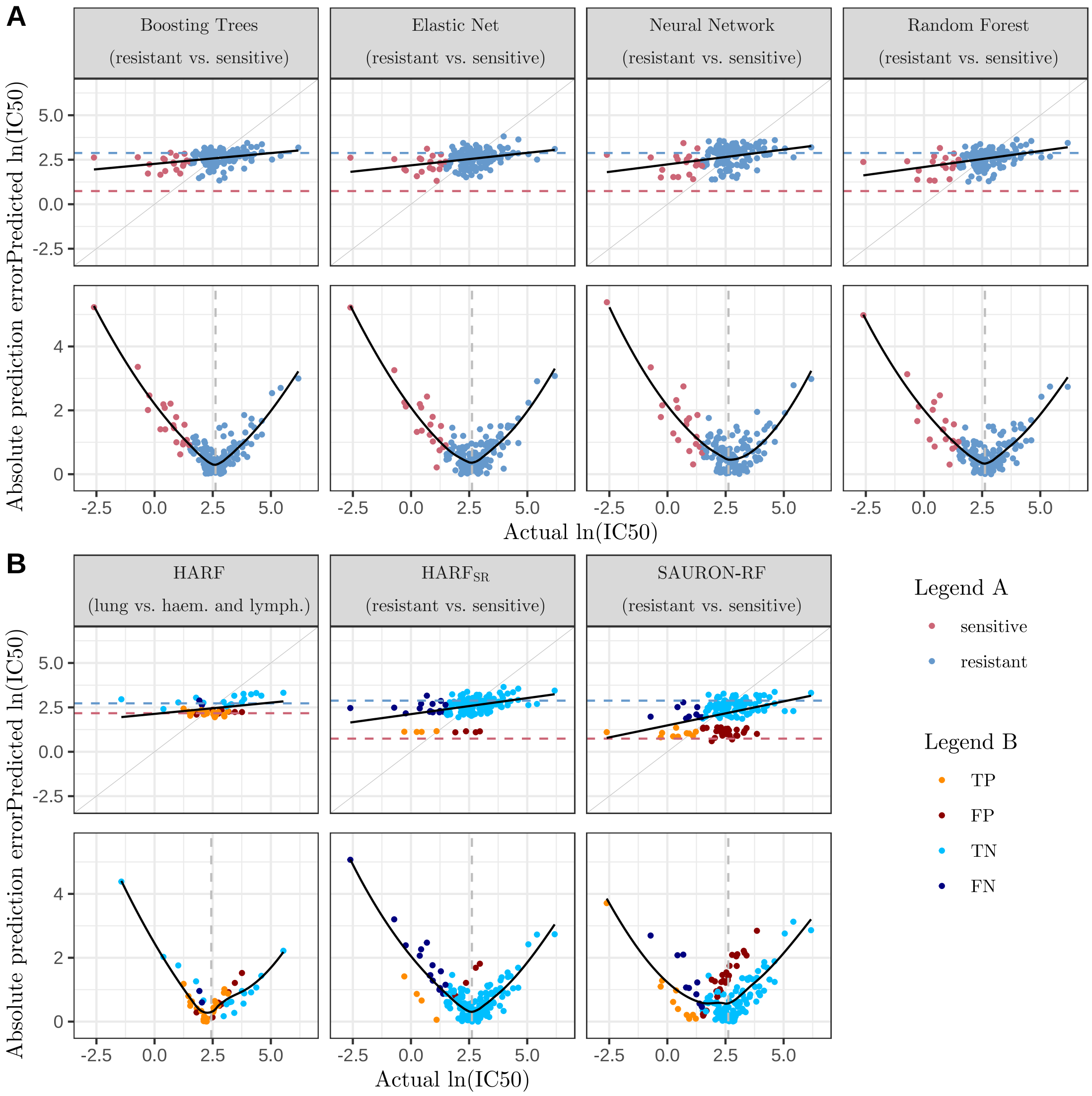

Figure 7: Regression performance of different ML methods for AZD3759. This figure exemplifies the performance of different ML algorithms when applied to the AZD3759 data set of the GDSC database using 20 input features. The upper rows of Fig. A and B show the predicted IC50 values plotted against the actual IC50 values including a fitted regression line, which is shown as a solid black line. The mean IC50 of training samples for each investigated class is depicted as a horizontal dashed line. The lower rows show the absolute prediction error. Here, the solid curve is a loess curve fitted to the error, the vertical dashed line gives the mean IC50 of all training samples. In Fig. A, we compare boosting trees, elastic net, neural networks and random forests. The point colouring indicates the class assignment (sensitive or resistant). The first plot in Fig. B depicts the performance of the original HARF algorithm applied to a restricted version of this data set containing only cell lines from two cancer types with different average drug responses, i.e., haematopoietic/lymphoid cell lines and lung cell lines. The second plot shows the performance of HARF when applied to our proposed class division ($HARF_{SR}$), and the last plot depicts the performance of the suggested SAURON-RF algorithm (SAURON-RF simple s.w., binary sens t.w.). Here, the point colouring represents the classification performance, i.e. we depict true positives (TP), false positives (FP), true negatives (TN), and false negatives (FN).

AZD4547

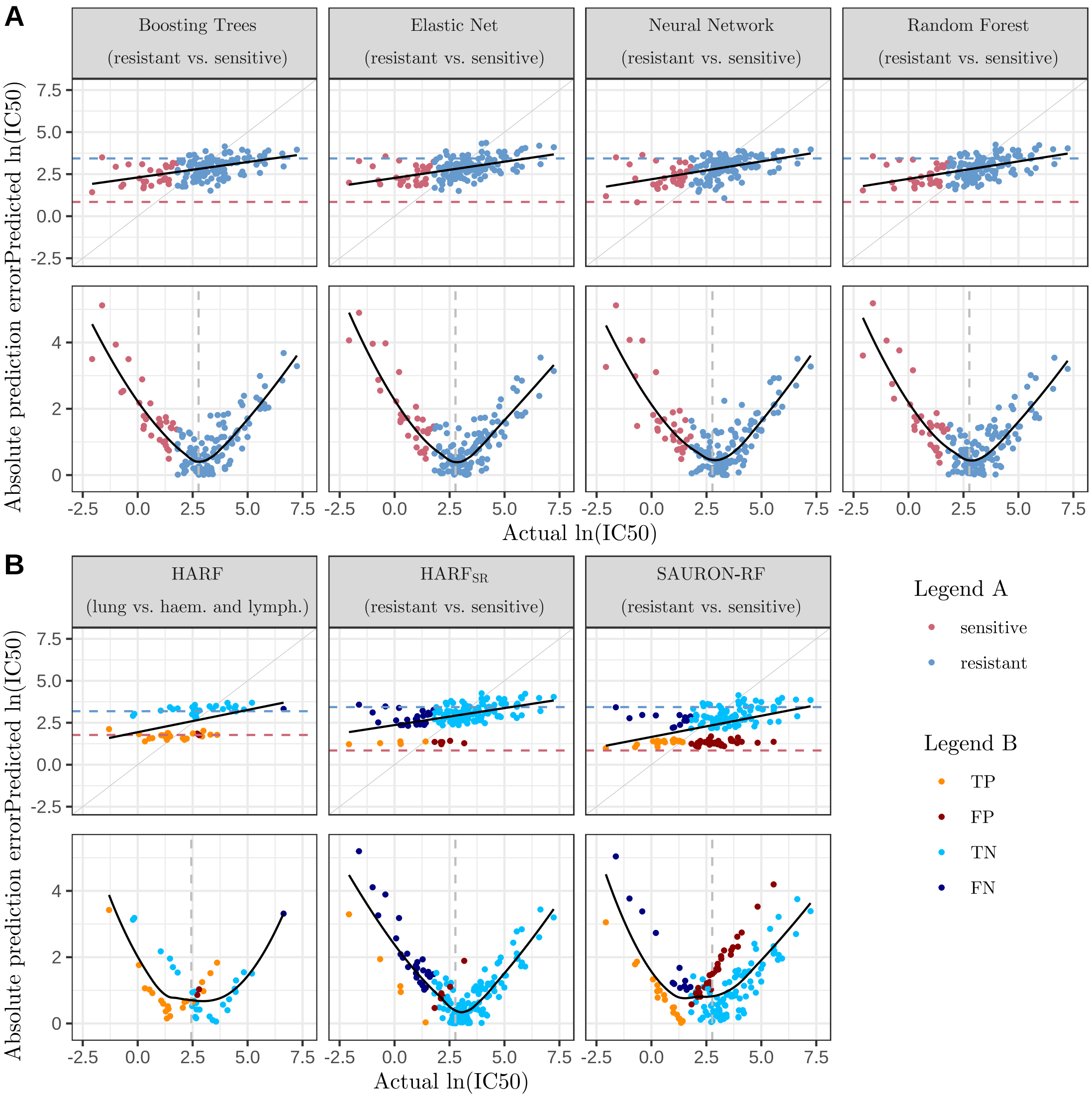

Figure 8: Regression performance of different ML methods for AZD4547. This figure exemplifies the performance of different ML algorithms when applied to the AZD4547 data set of the GDSC database using 20 input features. The upper rows of Fig. A and B show the predicted IC50 values plotted against the actual IC50 values including a fitted regression line, which is shown as a solid black line. The mean IC50 of training samples for each investigated class is depicted as a horizontal dashed line. The lower rows show the absolute prediction error. Here, the solid curve is a loess curve fitted to the error, the vertical dashed line gives the mean IC50 of all training samples. In Fig. A, we compare boosting trees, elastic net, neural networks and random forests. The point colouring indicates the class assignment (sensitive or resistant). The first plot in Fig. B depicts the performance of the original HARF algorithm applied to a restricted version of this data set containing only cell lines from two cancer types with different average drug responses, i.e., haematopoietic/lymphoid cell lines and lung cell lines. The second plot shows the performance of HARF when applied to our proposed class division ($HARF_{SR}$), and the last plot depicts the performance of the suggested SAURON-RF algorithm (SAURON-RF simple s.w., binary sens t.w.). Here, the point colouring represents the classification performance, i.e. we depict true positives (TP), false positives (FP), true negatives (TN), and false negatives (FN).

AZD5363

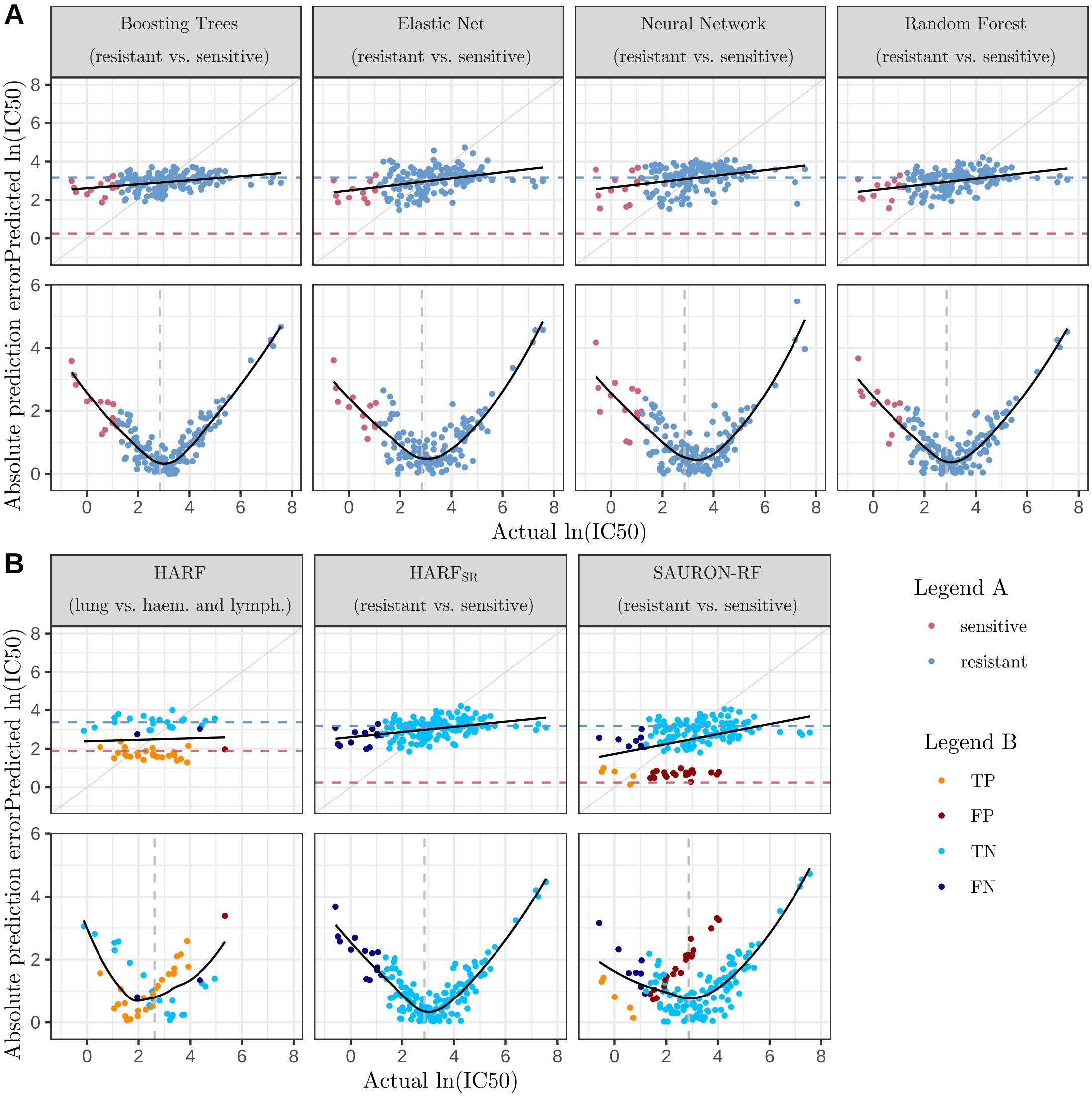

Figure 9: Regression performance of different ML methods for AZD5363. This figure exemplifies the performance of different ML algorithms when applied to the AZD5363 data set of the GDSC database using 20 input features. The upper rows of Fig. A and B show the predicted IC50 values plotted against the actual IC50 values including a fitted regression line, which is shown as a solid black line. The mean IC50 of training samples for each investigated class is depicted as a horizontal dashed line. The lower rows show the absolute prediction error. Here, the solid curve is a loess curve fitted to the error, the vertical dashed line gives the mean IC50 of all training samples. In Fig. A, we compare boosting trees, elastic net, neural networks and random forests. The point colouring indicates the class assignment (sensitive or resistant). The first plot in Fig. B depicts the performance of the original HARF algorithm applied to a restricted version of this data set containing only cell lines from two cancer types with different average drug responses, i.e., haematopoietic/lymphoid cell lines and lung cell lines. The second plot shows the performance of HARF when applied to our proposed class division (HARF_{SR}), and the last plot depicts the performance of the suggested SAURON-RF algorithm (SAURON-RF simple s.w., binary sens t.w.). Here, the point colouring represents the classification performance, i.e. we depict true positives (TP), false positives (FP), true negatives (TN), and false negatives (FN).

AZD6738

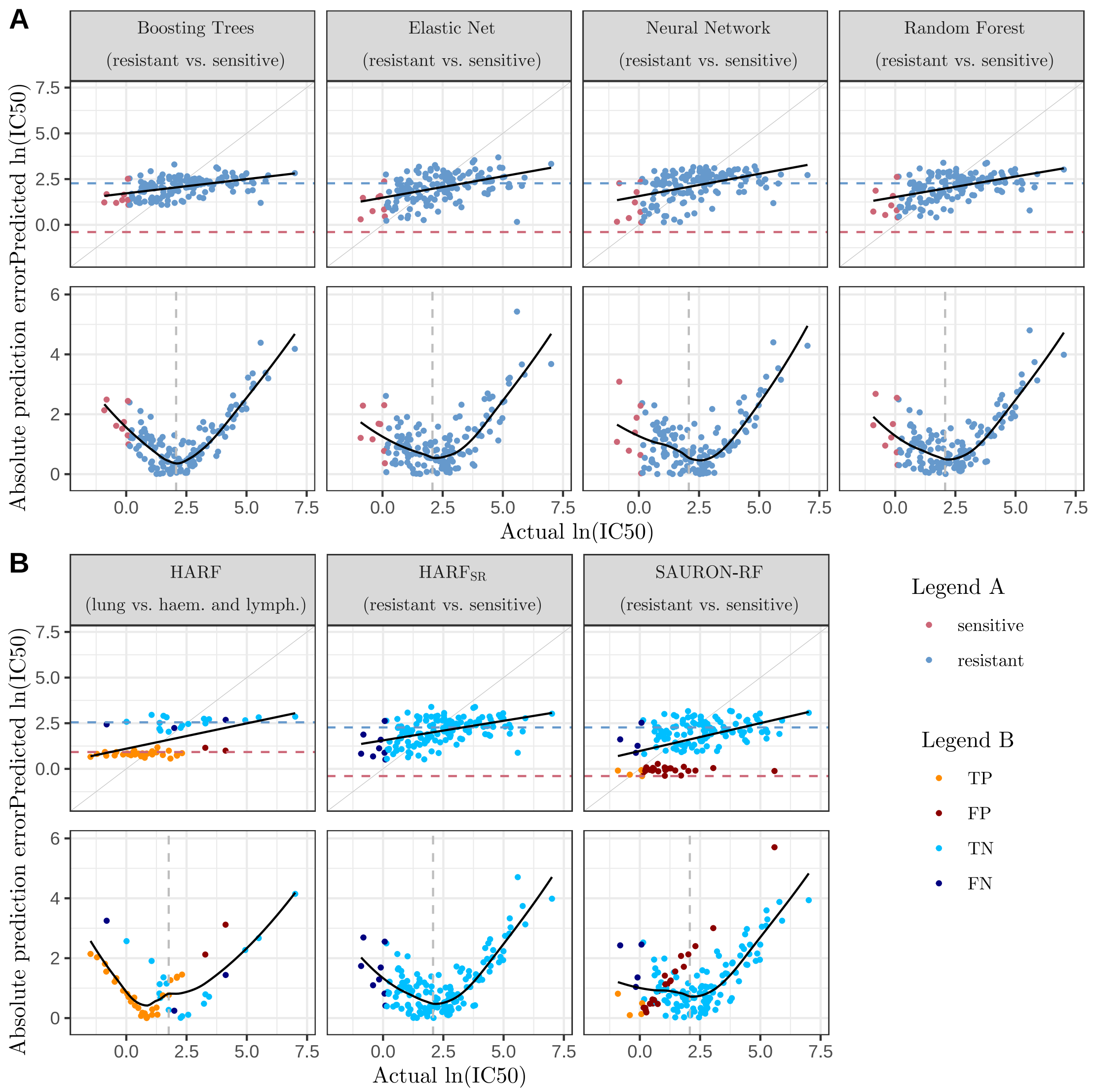

Figure 10: Regression performance of different ML methods for AZD6738. This figure exemplifies the performance of different ML algorithms when applied to the AZD6738 data set of the GDSC database using 20 input features. The upper rows of Fig. A and B show the predicted IC50 values plotted against the actual IC50 values including a fitted regression line, which is shown as a solid black line. The mean IC50 of training samples for each investigated class is depicted as a horizontal dashed line. The lower rows show the absolute prediction error. Here, the solid curve is a loess curve fitted to the error, the vertical dashed line gives the mean IC50 of all training samples. In Fig. A, we compare boosting trees, elastic net, neural networks and random forests. The point colouring indicates the class assignment (sensitive or resistant). The first plot in Fig. B depicts the performance of the original HARF algorithm applied to a restricted version of this data set containing only cell lines from two cancer types with different average drug responses, i.e., haematopoietic/lymphoid cell lines and lung cell lines. The second plot shows the performance of HARF when applied to our proposed class division (HARF$_{SR}$), and the last plot depicts the performance of the suggested SAURON-RF algorithm (SAURON-RF simple s.w., binary sens t.w.). Here, the point colouring represents the classification performance, i.e. we depict true positives (TP), false positives (FP), true negatives (TN), and false negatives (FN).

AZD7762

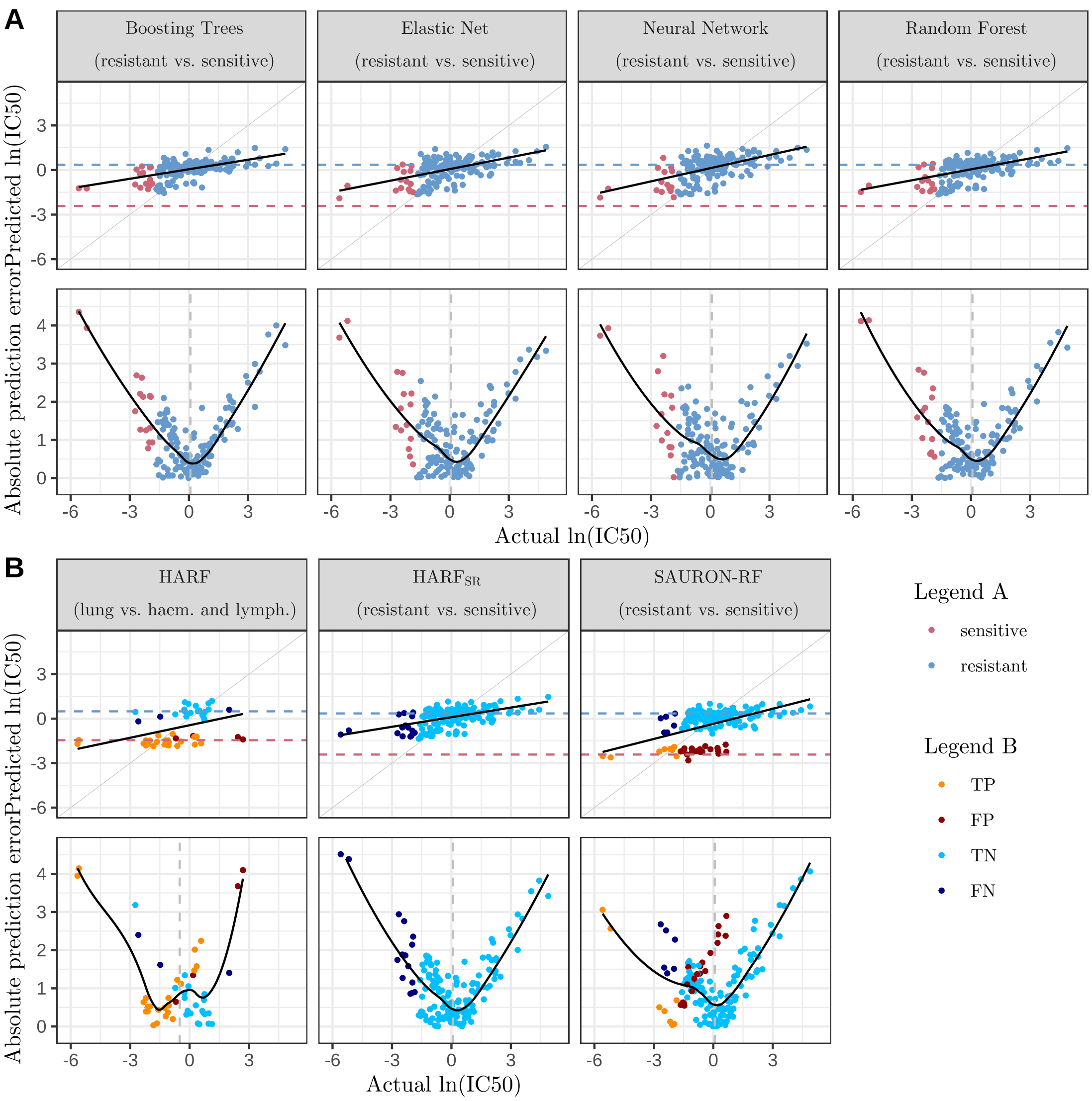

Figure 11: Regression performance of different ML methods for AZD7762. This figure exemplifies the performance of different ML algorithms when applied to the AZD7762 data set of the GDSC database using 20 input features. The upper rows of Fig. A and B show the predicted IC50 values plotted against the actual IC50 values including a fitted regression line, which is shown as a solid black line. The mean IC50 of training samples for each investigated class is depicted as a horizontal dashed line. The lower rows show the absolute prediction error. Here, the solid curve is a loess curve fitted to the error, the vertical dashed line gives the mean IC50 of all training samples. In Fig. A, we compare boosting trees, elastic net, neural networks and random forests. The point colouring indicates the class assignment (sensitive or resistant). The first plot in Fig. B depicts the performance of the original HARF algorithm applied to a restricted version of this data set containing only cell lines from two cancer types with different average drug responses, i.e., haematopoietic/lymphoid cell lines and lung cell lines. The second plot shows the performance of HARF when applied to our proposed class division (HARF$_{SR}$), and the last plot depicts the performance of the suggested SAURON-RF algorithm (SAURON-RF simple s.w., binary sens t.w.). Here, the point colouring represents the classification performance, i.e. we depict true positives (TP), false positives (FP), true negatives (TN), and false negatives (FN).

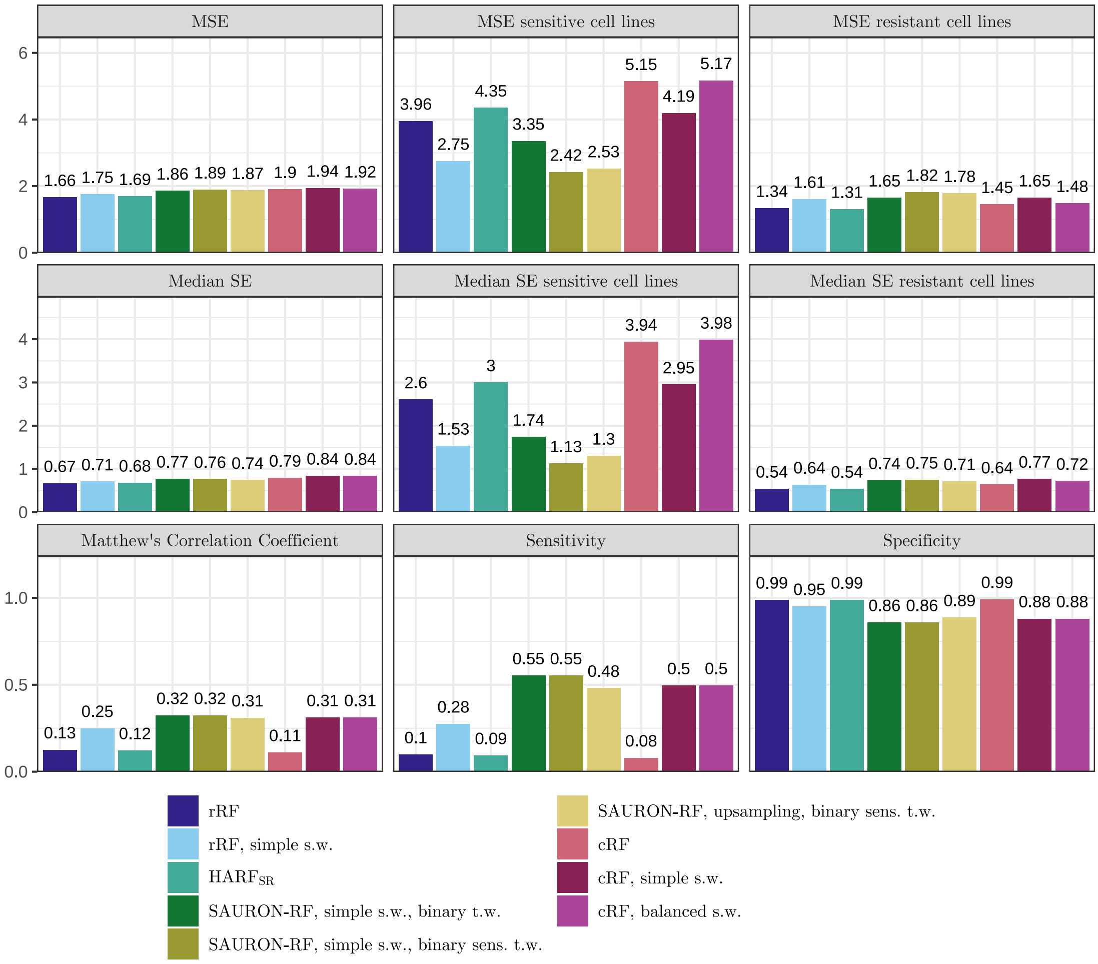

Figure 12: Random forest test set performance for 40 input features. In this figure, we compare regression random forests, classification random forests, and HARF with our suggested approach SAURON-RF. We show the average test set performance across the 86 different drugs for 40 input features.

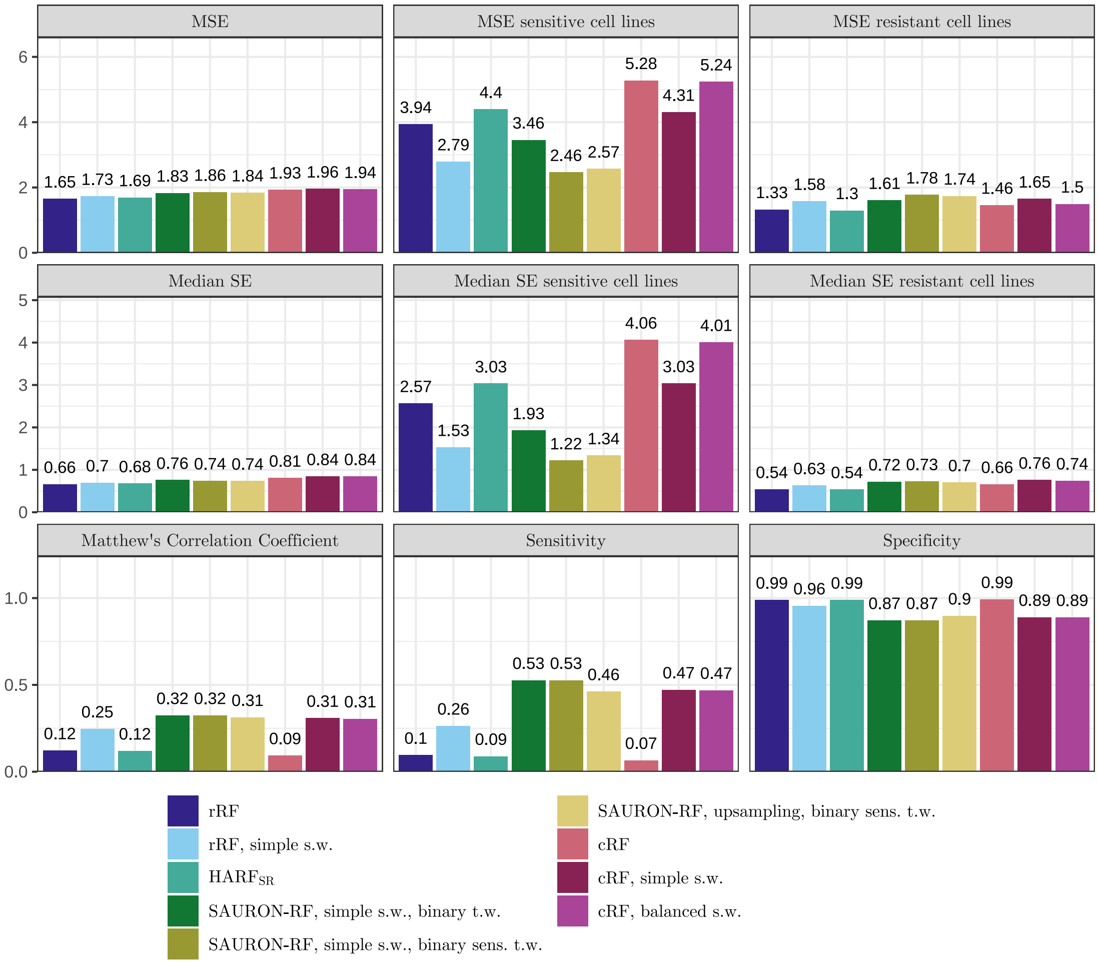

Figure 13: Random forest test set performance for 60 input features. In this figure, we compare regression random forests, classification random forests, and HARF with our suggested approach SAURON-RF. We show the average test set performance across the 86 different drugs for 60 input features.

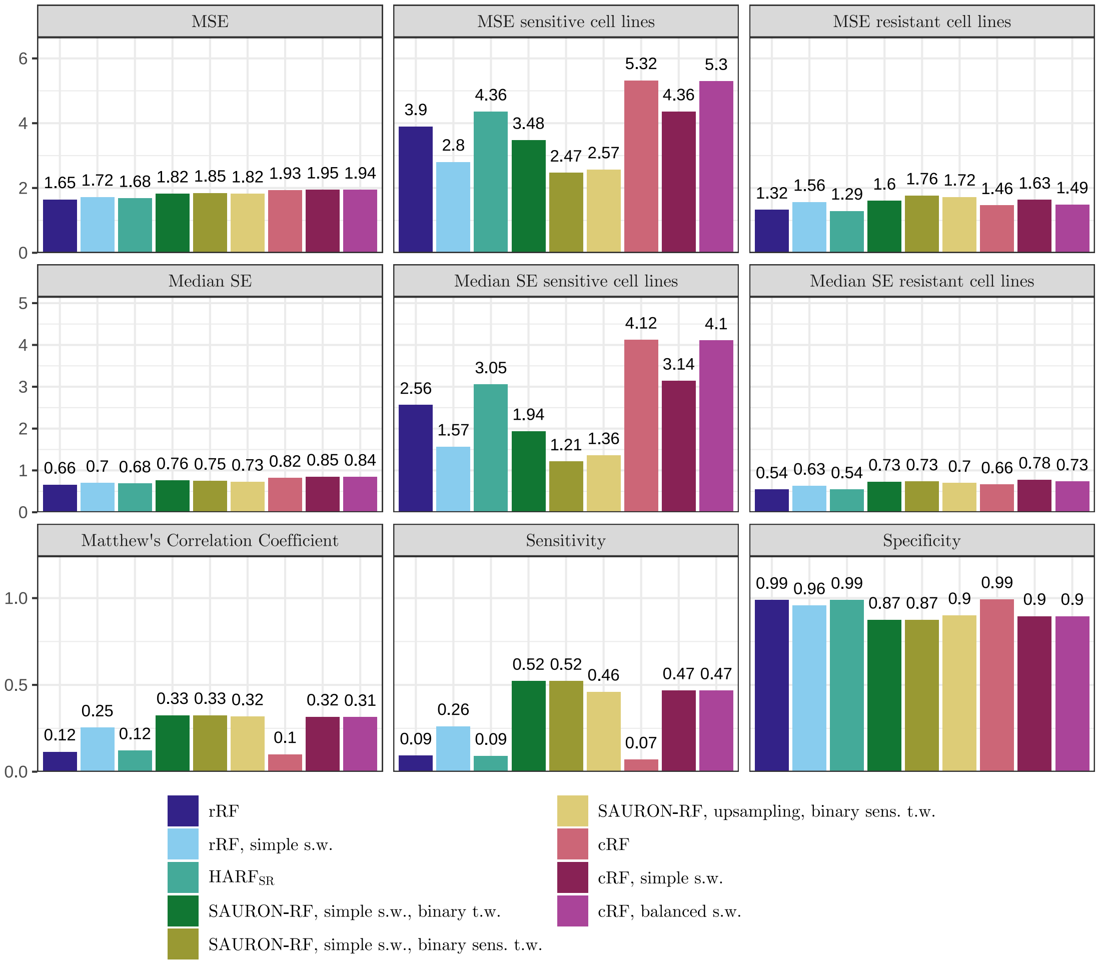

Figure 14: Random forest test set performance for 80 input features. In this figure, we compare regression random forests, classification random forests, and HARF with our suggested approach SAURON-RF. We show the average test set performance across the 86 different drugs for 80 input features.

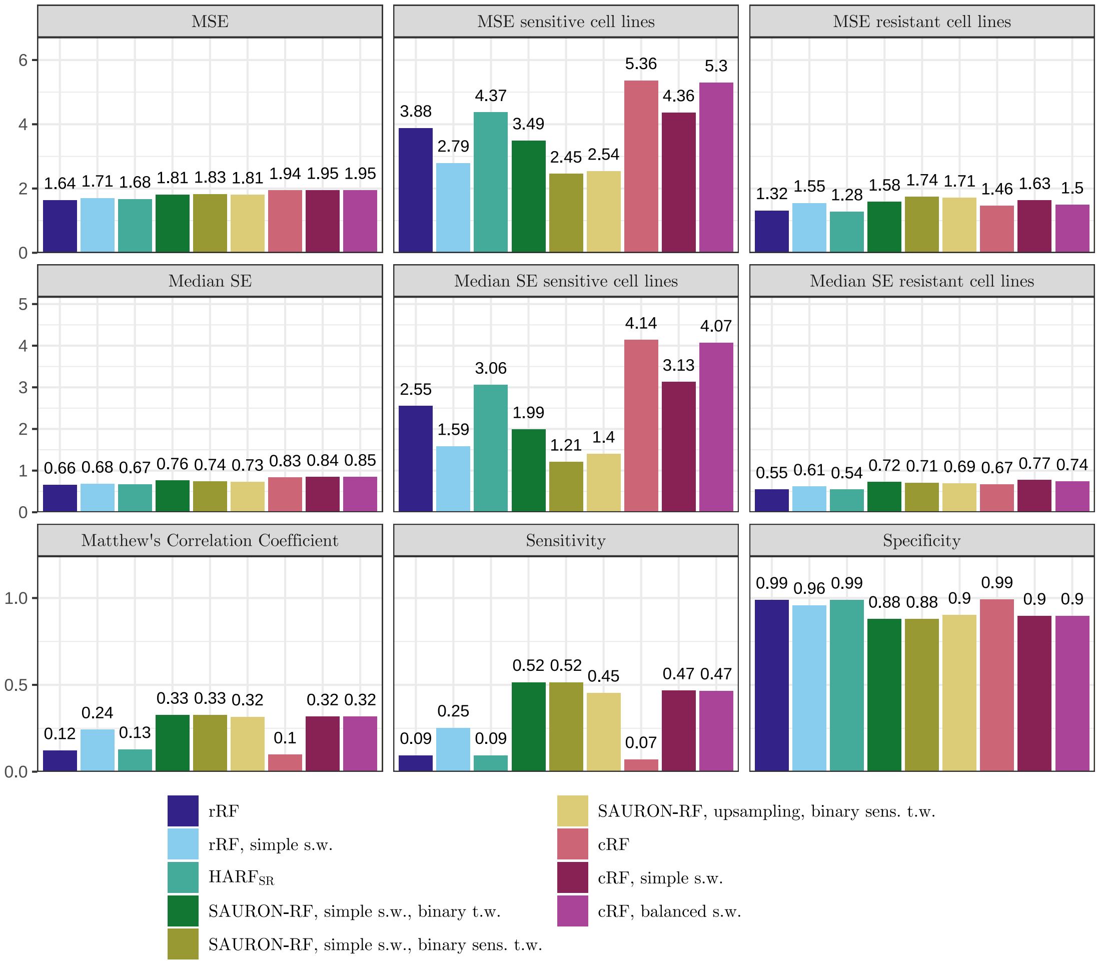

Figure 15: Random forest test set performance for 100 input features. In this figure, we compare regression random forests, classification random forests, and HARF with our suggested approach SAURON-RF. We show the average test set performance across the 86 different drugs for 100 input features.

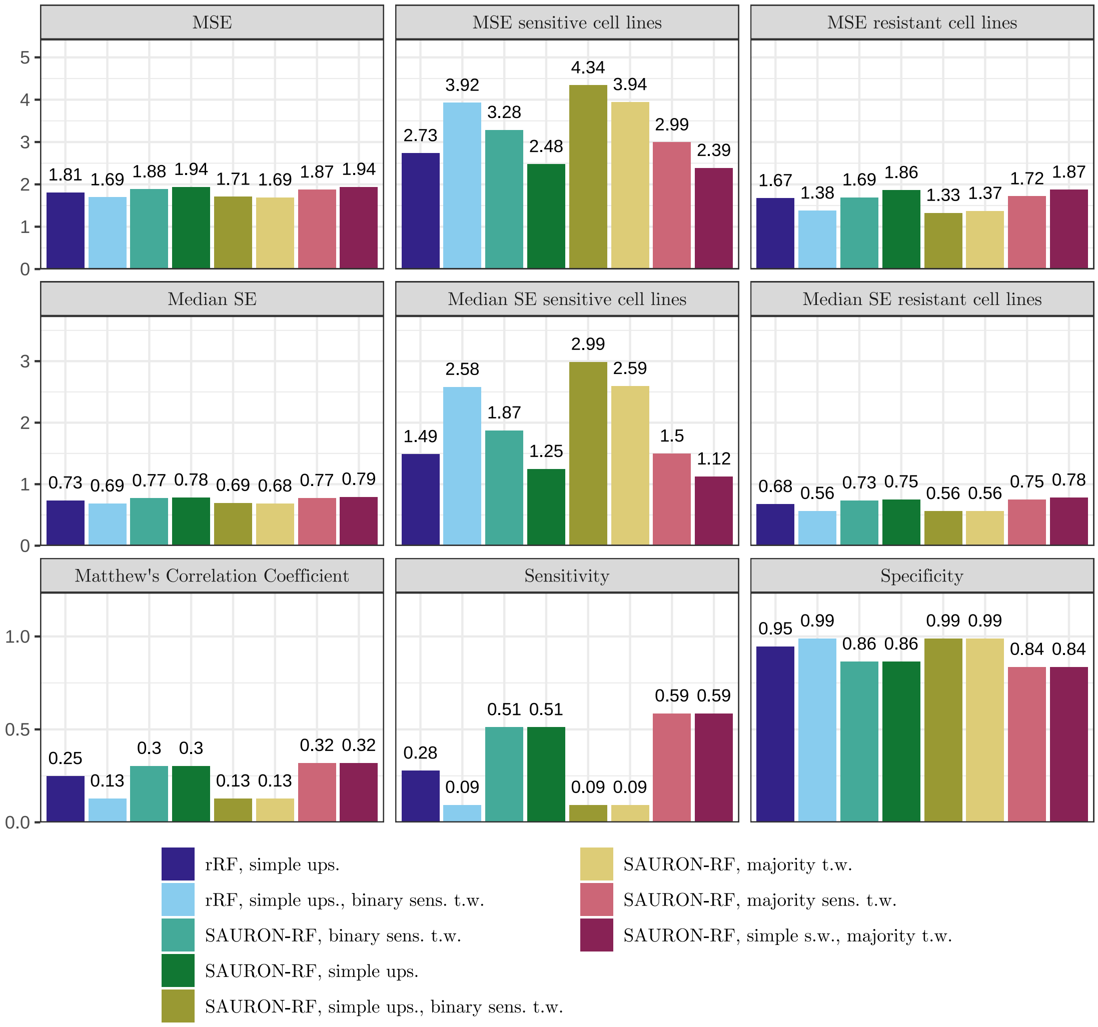

Figure 16: Test set performance for additional versions of rRF and SAURON-RF not discussed in the main text. We show the average test set performance across the 86 different drugs for 20 input features.

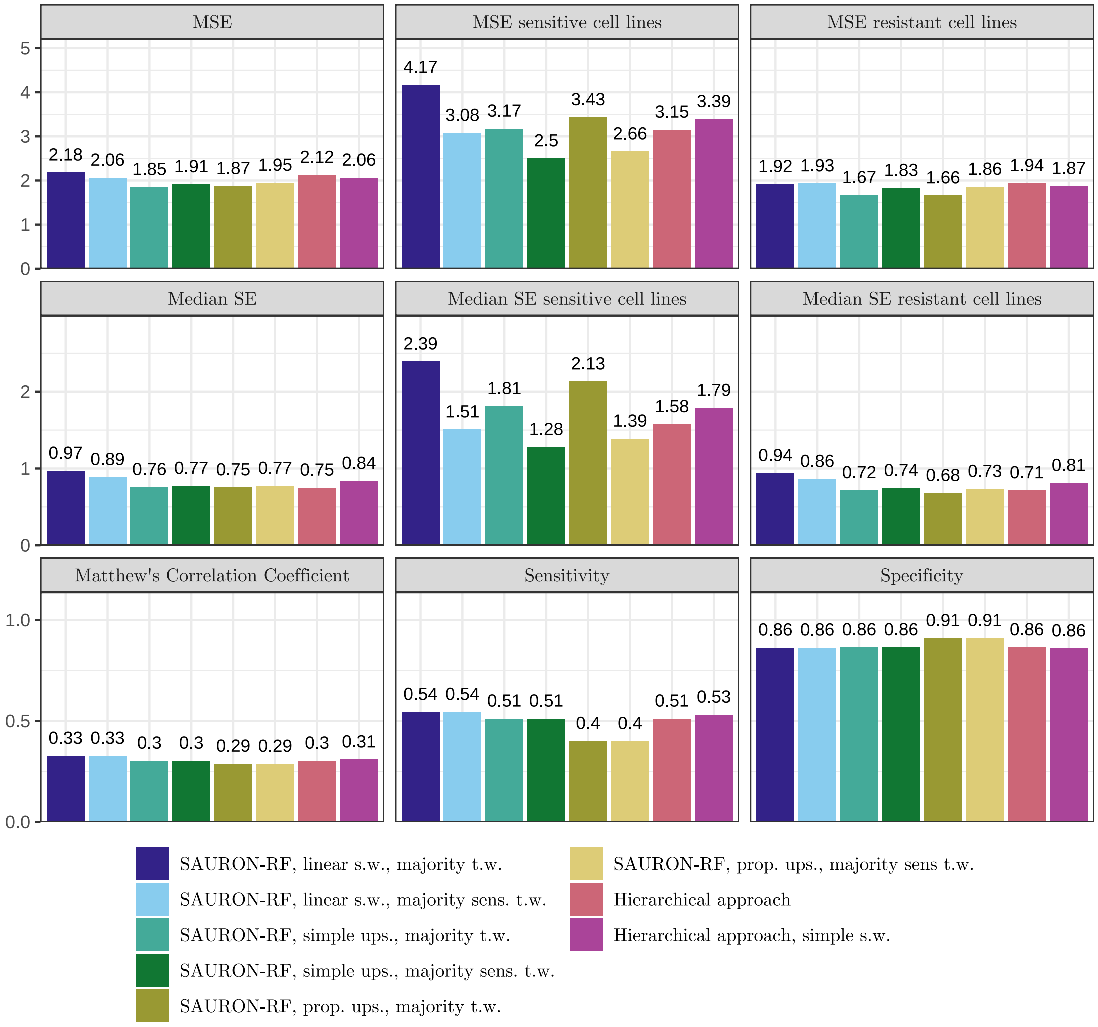

Figure 17: Test set performance for additional versions of SAURON-RF not discussed in the main text and for the hierarchical approach. We show the average test set performance across the 86 different drugs for 20 input features.

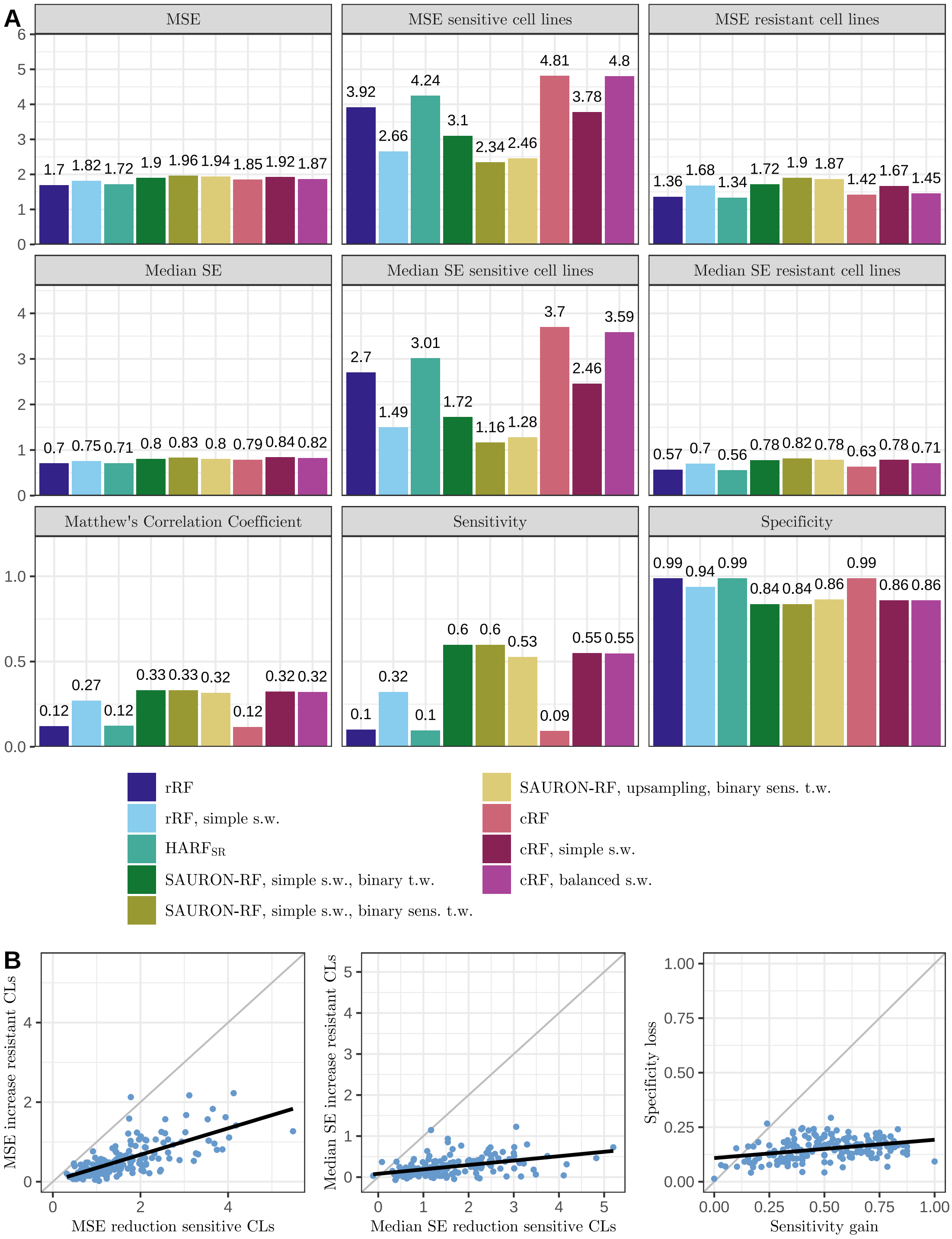

Figure 18: Random forest test set performance for 179 drugs. In Fig. A, we compare regression random forests (rRF), classification random forests (cRF), and HARF$_{SR}$ with different versions of our suggested approach SAURON-RF. We show the average test set performance across the 179 different drugs for 20 input features. In Fig. B, we depict for each of the 86 drugs the tradeoff between the absolute reduction in test error for the sensitive cell lines and the absolute increase in test error for the resistant cell lines when comparing our best-performing version of SAURON-RF (SAURON-RF simple s.w., binary sens. t.w.) with rRF.

3 Feature Importance

As described in the main text, we studied the feature importance of the top features for the best performing version of SAURON-RF. In Fig. 19 and 20 as well as Tables 1 to 10, we present the corresponding results.
Besides, we also investigated whether we are able to identify features that are more commonly associated with drug sensitivity or resistance. For each feature, we determine the number of drugs for which it is selected and then average its feature importance (see Fig. 21 to 30). In this way, we could identify three genes that are selected for many drugs and have a high average feature importance: PPIC, SDC4 and DCBLD2. All three genes encode transmembrane proteins. PPIC is involved in protein folding (Kumawat et al. (2020)), while SDC4 and DCBLD2 play a role in cellular signaling (Elfenbein and Simons (2013) and Xie et al. (2021)). DCBLD2 upregulation is associated with poor prognosis in various cancers including glioblastoma and colorectal cancer (Cheng et al. (2021); He et al. (2020); Xie et al. (2021)) and has recently been linked to 5-Fluorouracil resistance (Xie et al. (2021)). However, the role of these genes in multi-drug resistance remains to be investigated.

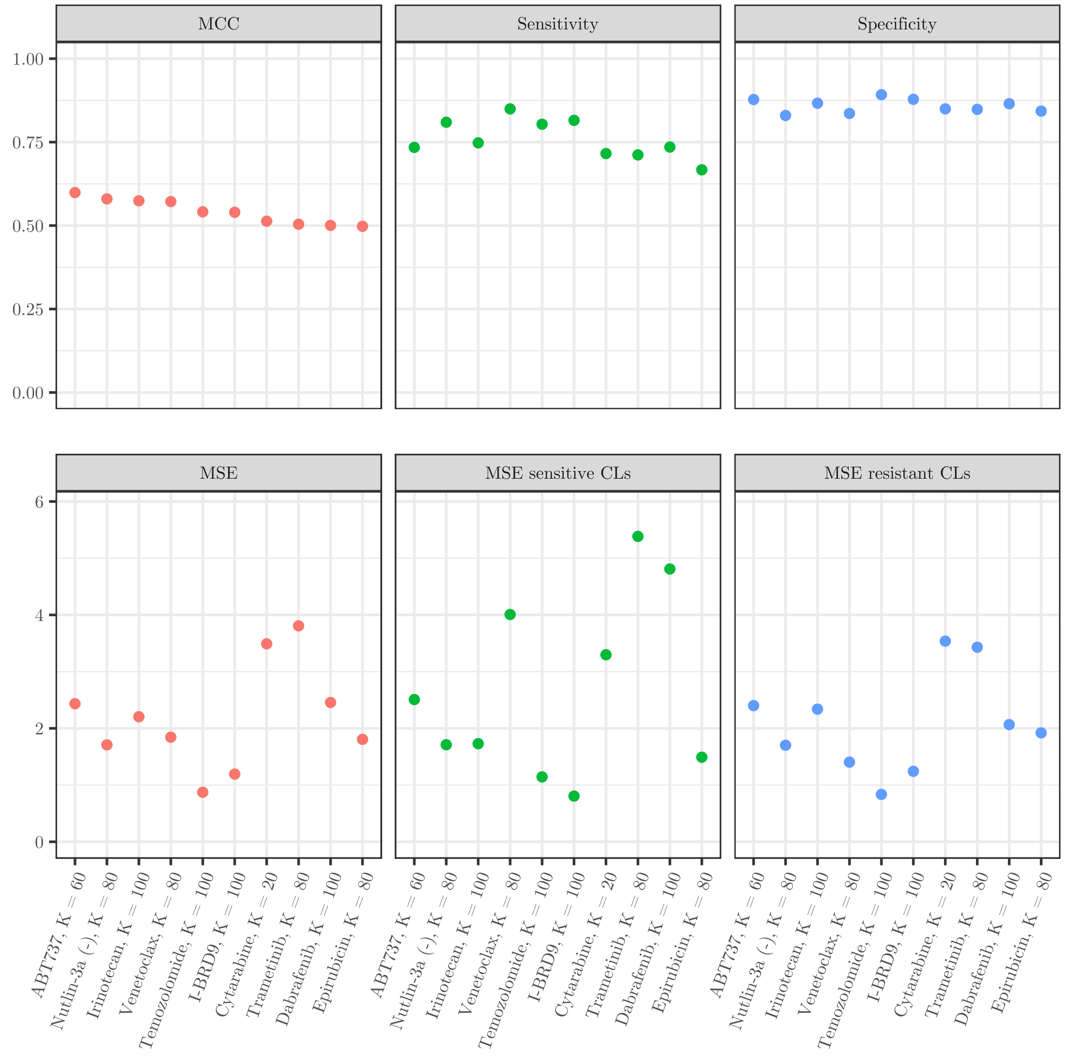

Figure 19: Top-performing drug data sets. This figure depicts the classification and regression performance for the 10 drugs with the highest CV MCC. We obtained this list by sorting the results for the best-performing SAURON-RF model (SAURON-RF, simple s.w., binary sens t.w.) for all drugs using the CV MCC. In Fig. 20, we depict the corresponding feature importances.

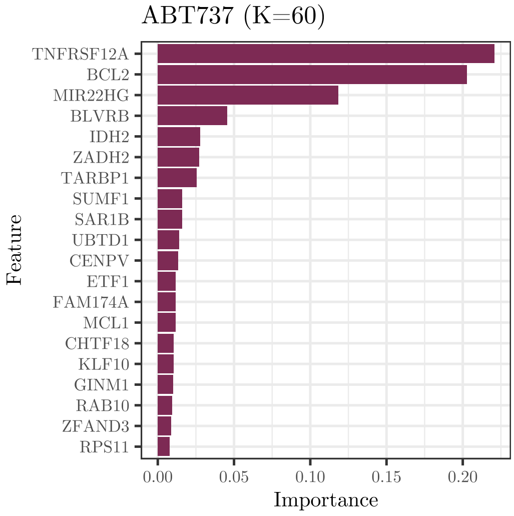

(a) **ABT737**

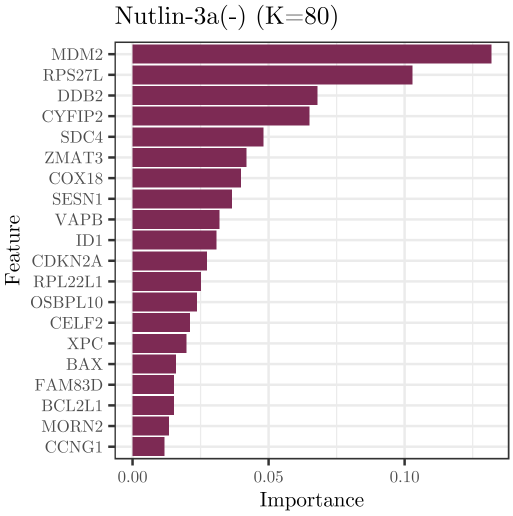

(b) **Nutlin-3a(-)**

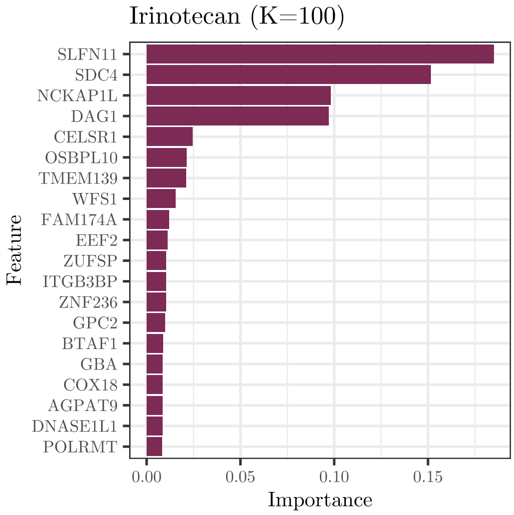

(c) **Irinotecan**

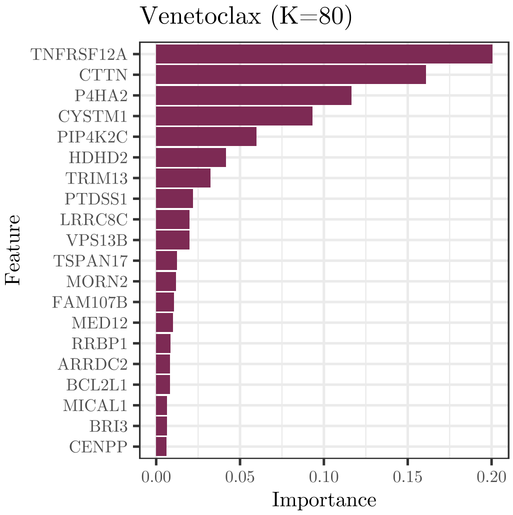

(d) **Venetoclax**

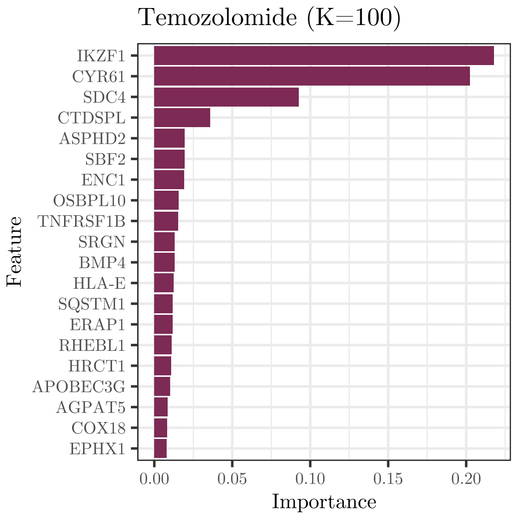

(e) **Temozolomide**

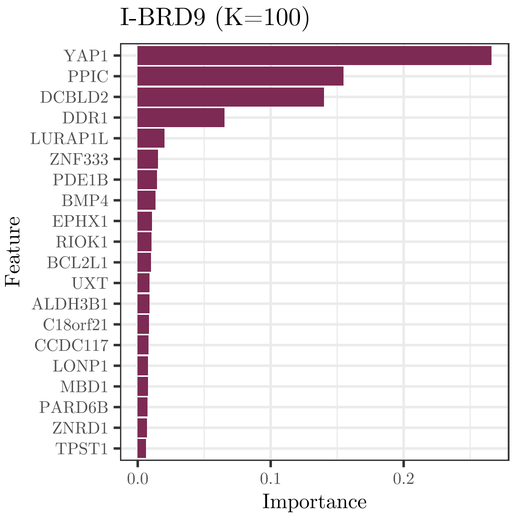

(f) **I-BRD9**

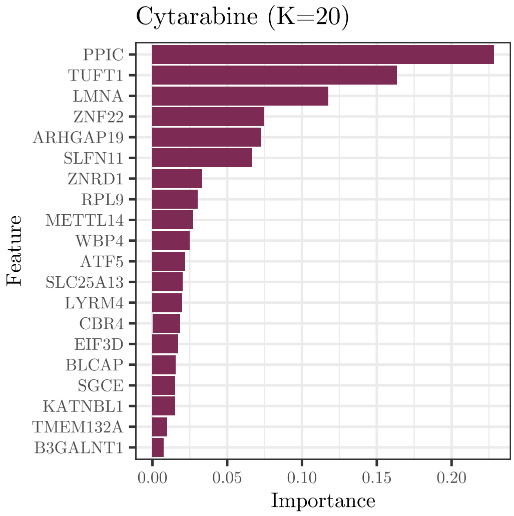

(g) **Cytarabine**

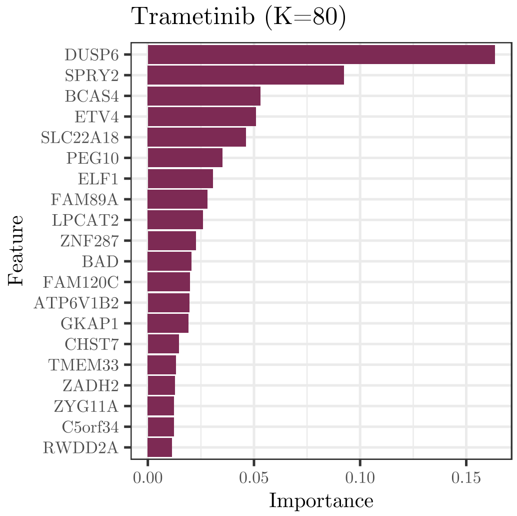

(h) **Trametinib**

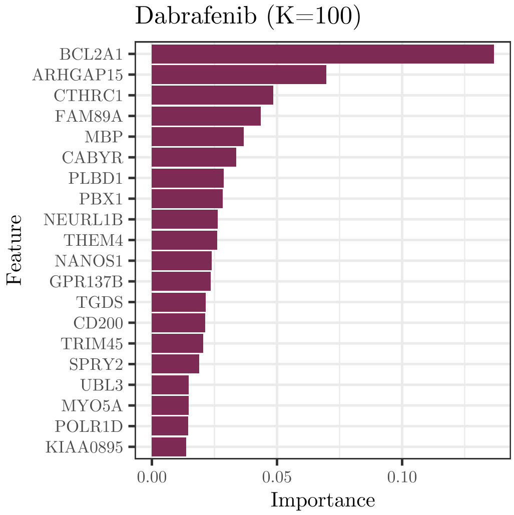

(i) **Dabrafenib**

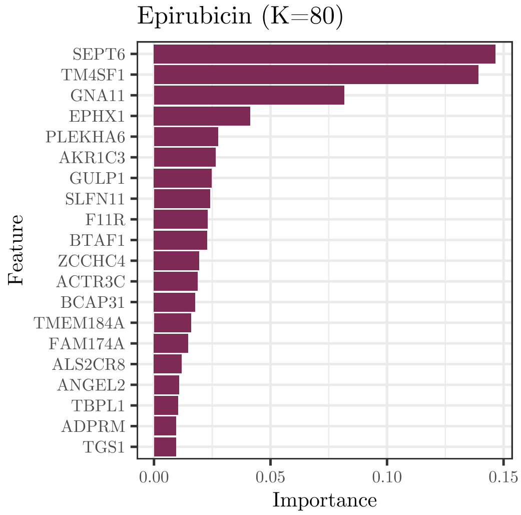

(j) **Epirubicin**

Figure 20: In Sub-Fig. (a - j), we present the feature importance of the 10 drugs with the highest CV MCC in our experiments for the best-performing SAURON-RF model (SAURON-RF, simple s.w. binary sens t.w.). In Table 1 to 10, we summarize the functions for the top 5 features of each drug and give literature evidence for implication in drug response if applicable.

Drug: ABT737
Target(s): BCL2, BCL-XL, BCL-W, BCL-B, BFL1
Target pathway: Apoptosis regulation

Feature	Feature function (GeneCards Safran et al. (2010))	Validated?
TNFRSF12A	involved in extrinsic apopotosis and wound healing regulation	✓ Whitsett et al. (2014)
BCL2	located in the outer mitochondrial membrane and involved in apoptosis	✓ drug target
MIR22HG	involved in wound response	(✓) involved in downregulation of BCL2 (Zhang et al. (2020); Pasqualini et al. (2015))
BLVRB	catalyzes final step of heme metabolism	(✓) low expression associated with obatoclax sensitivity (also BCL2 inhibitor) (Rees et al. (2016))
IDH2	catalyzes the oxidative decarboxylation of isocitrate to 2-oxoglutarat	✓ mutations associated with increased sensitivity (Chan et al. (2015); Rahmani et al. (2015))

Table 1: This table lists the five features with highest importance in the prediction model for ABT737 using our best-performing SAURON-RF version with K=60. Additionally, the drug target(s) and target pathway (both derived from the GDSC) are shown.

Drug: Nutlin-3a(-)
Target(s): MDM2
Target pathway: p53 pathway

Feature	Feature function (GeneCards Safran et al. (2010))	Validated?
MDM2	nuclear-localized E3 ubiquitin ligase, which can promote tumour formation	✓ drug target
RPS27L	might be a component of the 40S ribosomal subunit	✓ Pishas et al. (2014)
DDB2	part of protein complex that is involved in nucleotide excision repair and cellular response to DNA damage in general	(✓) maybe yes, has to do with MDM2 and nucleotide excision repair Stoyanova et al. (2009), Nutlin-3a(-) treatment increases DDB2 expression substantially (Zanjirband et al. (2016))
CYFIP2	participates in T-cell adhesion and p53-dependent induction of apoptosis	(✓) Nutlin-3a(-) treatment increases CYFIP2 expression substantially (Kumamoto et al. (2008))
SDC4	is a transmembrane proteoglycan involved in intracellular signaling	×

Table 2: This table lists the five features with highest importance in the prediction model for Nutlin-3a(-) using our best-performing SAURON-RF version with K=80. Additionally, the drug target(s) and target pathway (both derived from the GDSC) are shown.

Drug: Irinotecan
Target(s): TOP1
Target pathway: DNA replication

Feature	Feature function (GeneCards Safran et al. (2010))	Validated?
SLFN11	involved in tRNA binding, defense response to virus, negative regulation of G1/S transition and replication fork arrest	✓ Coussy et al. (2020)
SDC4	is a transmembrane proteoglycan involved in intracellular signaling	(✓) high plasma levels of SDC1 related to Irinotecan resistance (Krushkal et al. (2017))
NCKAP1L	transmembrane protein, part of the WAVE complex that regulates cell shape, expressed in haematopoietic cells only	×
DAG1	part of complex that links extracellular matrix to cytoskeleton in skeletal muscle	(✓) combination treatment with PHY906 reduces DAG1 expression compared to other treatments (Xing et al. (2020))
CELSR1	it is postulated that the corresponding protein acts as receptor involved in contact-mediated communication	×

Table 3: This table lists the five features with highest importance in the prediction model for Irinotecan using our best-performing SAURON-RF version with K=100. Additionally, the drug target(s) and target pathway (both derived from the GDSC) are shown.

Drug: Venetoclax
Target(s): BCL2
Target pathway: Apoptosis regulation

Feature	Feature function (GeneCards Safran et al. (2010))	Validated?
TNFRSF12A	involved in extrinsic apopotosis and wound healing regulation	×
CTTN	involved in actin cytoskeleton and cell shape regulation	×
P4HA2	involved in collagen synthesis and amino acid metabolism	×
CYSTM1	related to the innate immune system	×
PIP4K2C	kinase	×

Table 4: This table lists the five features with highest importance in the prediction model for Venetoclax using our best-performing SAURON-RF version with K=80. Additionally, the drug target(s) and target pathway (both derived from the GDSC) are shown.

Drug: Temozolomide
Target(s): DNA alkylating agent
Target pathway: DNA replication

Feature	Feature function (GeneCards Safran et al. (2010))	Validated?
IKZF1	transcription factor	✓ Iacobucci et al. (2012)
CYR61	besides others involved in cell proliferation, chemotaxis and angiogenesis	✓ Tan et al. (2018)
SDC4	involved in intracellular signaling	×
CTDSPL	has phosphatase activity	✓ host gene of miR-26a, whose overexpression correlates with poor treatment prognosis (Ge et al. (2018))
ASPHD2	has metal ion binding activity	×

Table 5: This table lists the five features with highest importance in the prediction model for Temozolomide using our best-performing SAURON-RF version with K=100. Additionally, the drug target(s) and target pathway (both derived from the GDSC) are shown.

Drug: I-BRD9
Target(s): BRD9
Target pathway: Chromatin other

Feature	Feature function (GeneCards Safran et al. (2010))	Validated?
YAP1	transcription factor, tumor suppressor	×
PPIC	protein folding	×
DCBLD2	negative regulation of cell growth	×
DDR1	transferase activity	×
LURAP1L	involved in positive regulation of I-kappaB kinase/NF-kappaB signaling	×

Table 6: This table lists the five features with highest importance in the prediction model for I-BRD9 using our best-performing SAURON-RF version with K=100. Additionally, the drug target(s) and target pathway (both derived from the GDSC) are shown.

Drug: Cytarabine
Target(s): Antimetabolite
Target pathway: Other

Feature	Feature function (GeneCards Safran et al. (2010))	Validated?
PPIC	protein folding	×
TUFT1	structural constituent of tooth enamel	×
LMNA	part of nuclear lamina	×
ZNF22	transcription factor	×
ARHGAP19	has GTPase activator activity	×

Table 7: This table lists the five features with highest importance in the prediction model for Cytarabine using our best-performing SAURON-RF version with K=20. Additionally, the drug target(s) and target pathway (both derived from the GDSC) are shown.

Drug: Trametinib
Target(s): MEK1, MEK2
Target pathway: ERK MAPK signaling

Feature	Feature function (GeneCards Safran et al. (2010))	Validated?
DUSP6	has phosphatase activity	✓ Wu et al. (2018)
SPRY2	invovled in kinase binding	✓ Schreck et al. (2020)
BCAS4	part of BLOC-1 complex and associated with breast cancer	×
ETV4	transcription factor	✓ Da Vià et al. (2020)
SLC22A18	has transporter activity	×

Table 8: This table lists the five features with highest importance in the prediction model for Trametinib using our best-performing SAURON-RF version with K=80. Additionally, the drug target(s) and target pathway (both derived from the GDSC) are shown.

Drug: Dabrafenib
Target(s): BRAF
Target pathway: ERK MAPK signaling

Feature	Feature function (GeneCards Safran et al. (2010))	Validated?
BCL2A1	involved in apoptosis	✓ Flaherty et al. (2015)
ARHGAP15	involved in RHO GTPase regulation	×
CTHRC1	may be involved in wound healing	✓ Eriksson et al. (2016)
FAM89A	-	×
MBP	involved in formation and stabilization of myelin membrane	✓ Li et al. (2014)

Table 9: This table lists the five features with highest importance in the prediction model for Dabrafenib using our best-performing SAURON-RF version with K=100. Additionally, the drug target(s) and target pathway (both derived from the GDSC) are shown.

Drug: Epirubicin
Target(s): Anthracycline
Target pathway: DNA replication

Feature	Feature function (GeneCards Safran et al. (2010))	Validated?
SEPT6	involved in actin cytoskeleton organization	×
TM4SF1	involved in cell growth and motility	×
GNA11	involved in transmembrane signaling	×
EPHX1	enzyme	×
PLEKHA6	involved in metabolism	×

Table 10: This table lists the five features with highest importance in the prediction model for Epirubicin using our best-performing SAURON-RF version with K=80. Additionally, the drug target(s) and target pathway (both derived from the GDSC) are shown.

Model	Parameter	Value(s)
Boosting Trees	n.trees	100
	interaction.depth	4
	shrinkage	0.1
	bag.fraction	0.5
	distribution	"gaussian"
	cv.folds	5
Elastic Net	alpha	$[0, 1]$
	lambda	10^v, $v \in [-2, 2]$
	standardize	TRUE
	CV folds	5
Neural Network	Loss function	MSE
	Optimizer	Adam
	Learning rate	0.001
	# Hidden layers	1, 2, 3
	# Nodes per hidden layer	same as input layer
	Activation function	tanh (none in output layer)
	Weight initialization	Glorot uniform
	Bias initialization	0.01
	Weight regularization	L2
	Bias regularization	none
	Dropout	10%
	Batch size	128
	Epochs	max. 4000 (early stopping)
	Patience	15 epochs
	Data fraction for validation	20%

Table 11: Summary of all model parameters used to fit boosting trees, elastic nets and neural networks for the generation of the results depicted in Fig. 2 in the main text and Fig. 1 of this Supplement.

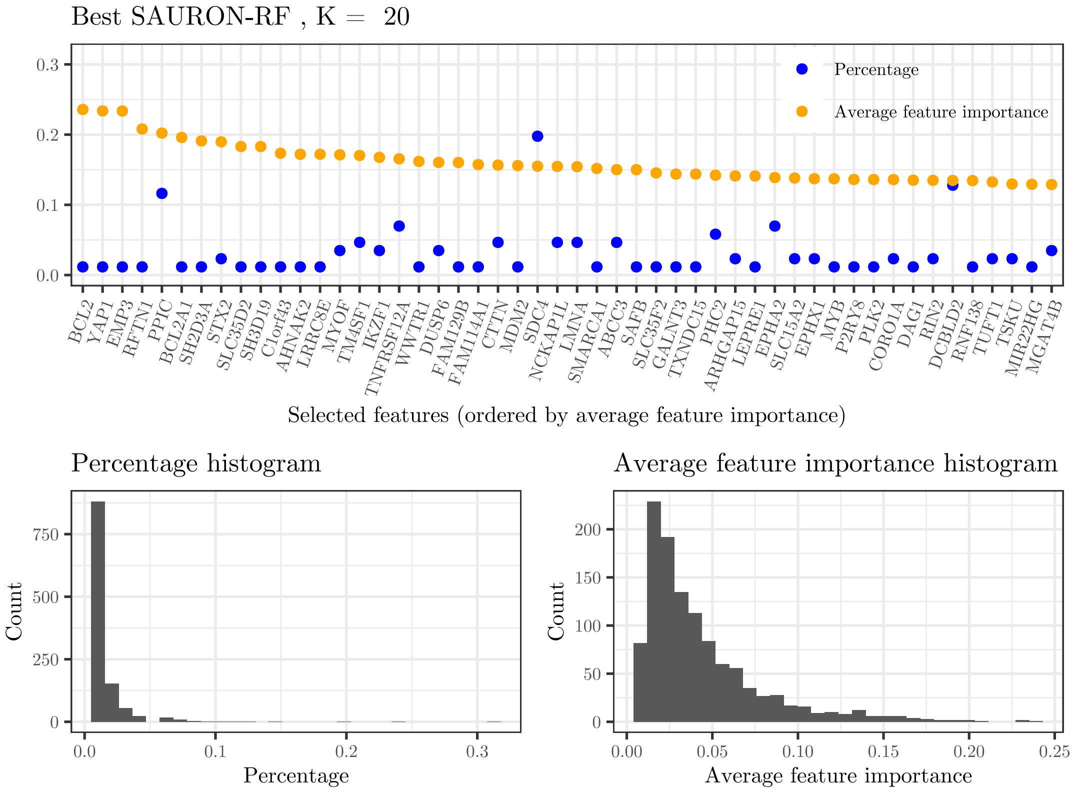

Figure 21: Feature importance for SAURON-RF using 20 input features (sorted by feature importance). This figure shows the 50 features with the highest average feature importance for the best-performing SAURON-RF model using $K = 20$ input features per drug. The per feature average was calculated based on the drugs for which this feature was selected in the feature selection. The corresponding percentage of drugs is also depicted (blue dots).

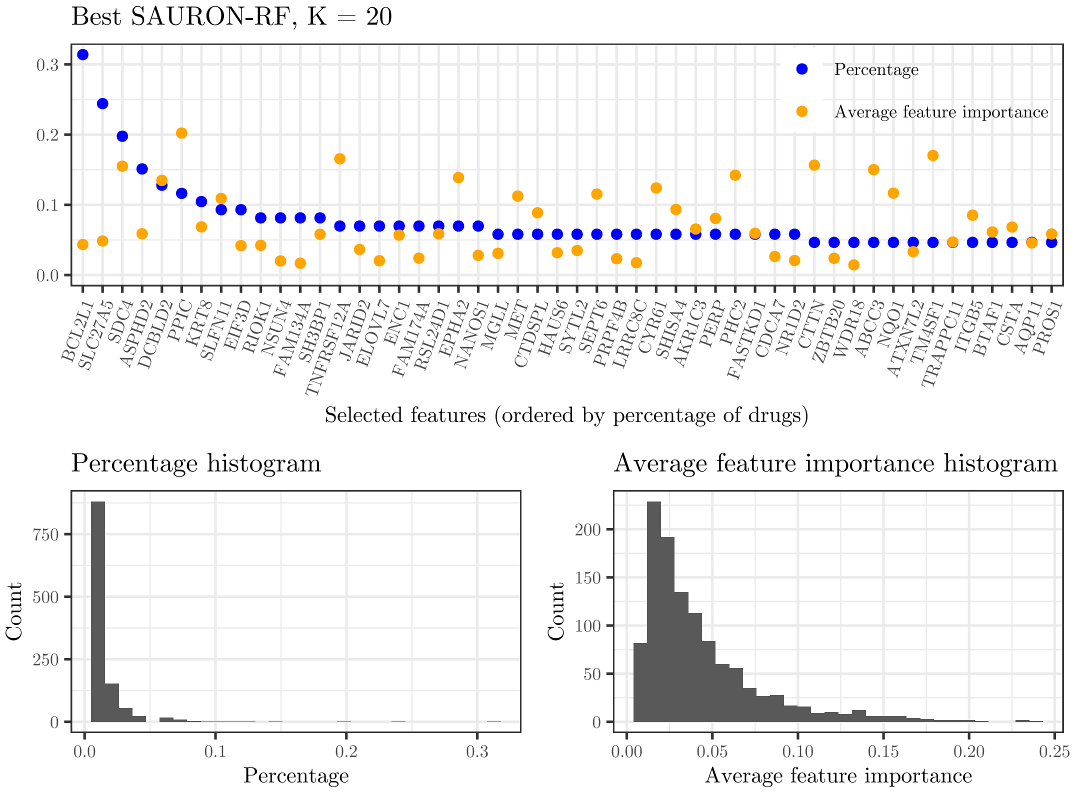

Figure 22: Feature importance for SAURON-RF using 20 input features (sorted by percentage of drugs affected). This figure shows the features sorted decreasingly by the percentage of drugs for which they were selected when using $K = 20$ input features per drug. In addition, the average feature importance is shown (yellow dots). The per feature average was calculated based on the drugs for which this feature was selected in the feature selection.

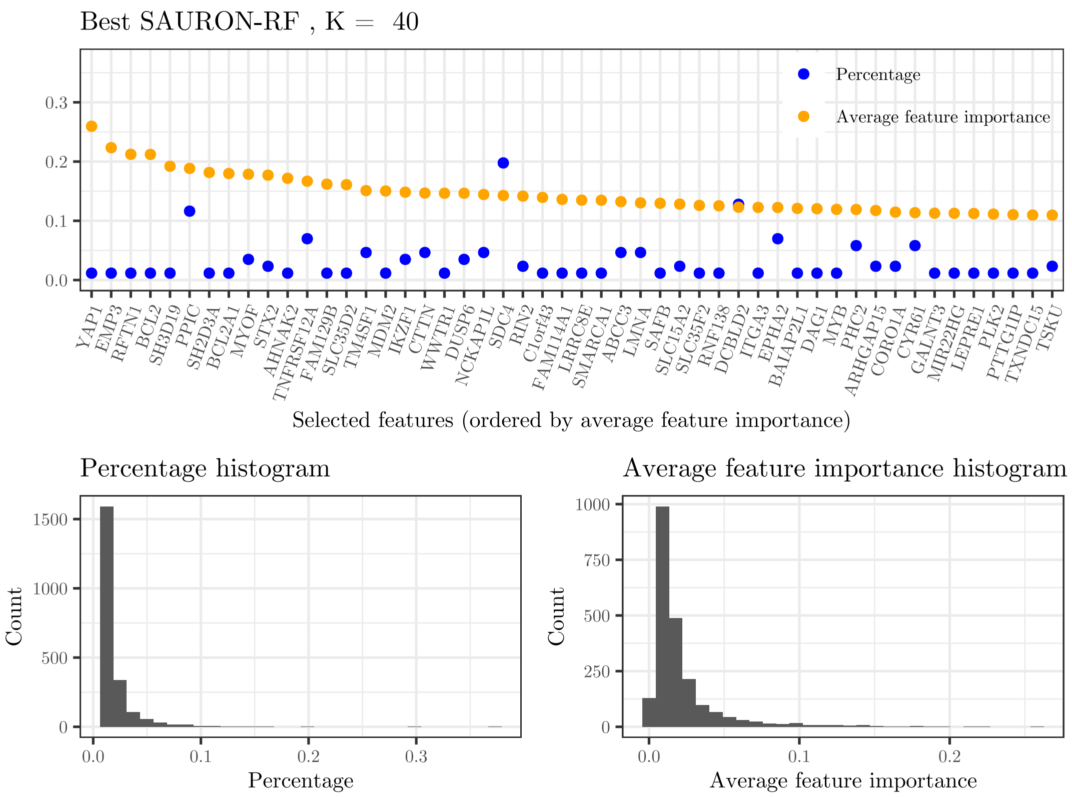

Figure 23: Feature importance for SAURON-RF using 40 input features (sorted by feature importance). This figure shows the 50 features with the highest average feature importance for the best-performing SAURON-RF model using $K = 40$ input features per drug. The per feature average was calculated based on the drugs for which this feature was selected in the feature selection. The corresponding percentage of drugs is also depicted (blue dots).

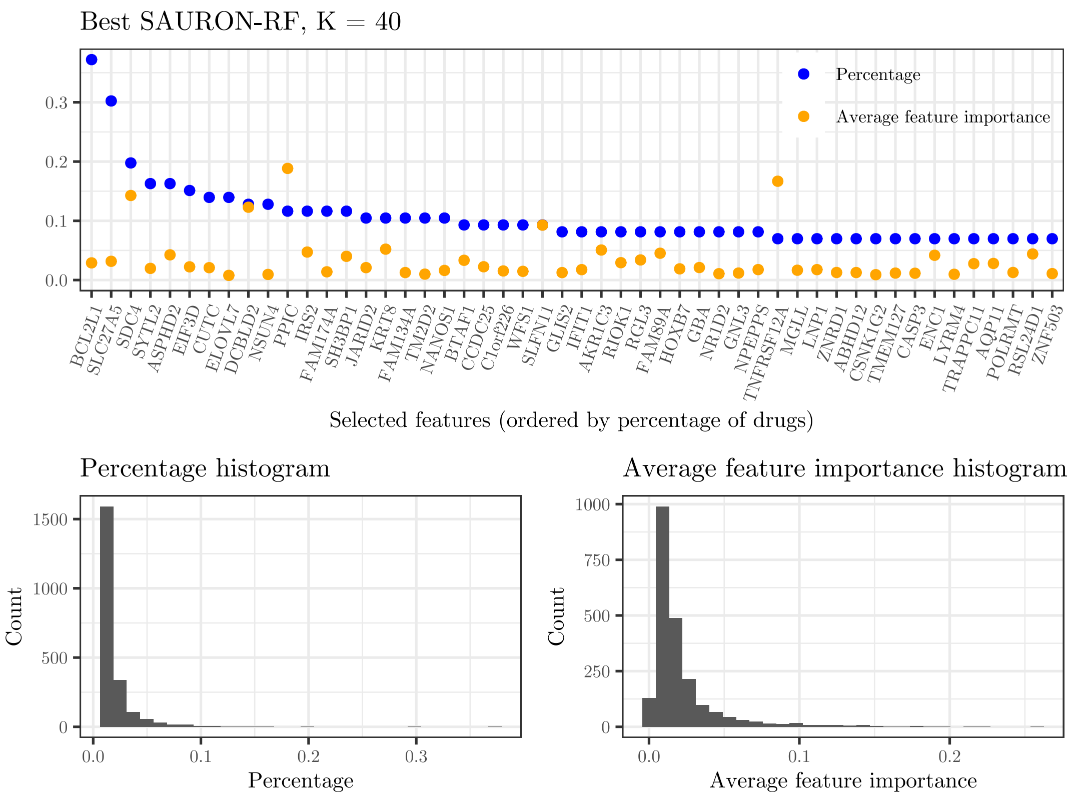

Figure 24: Feature importance for SAURON-RF using 40 input features (sorted by percentage of drugs affected). This figure shows the features sorted decreasingly by the percentage of drugs for which they were selected when using $K = 40$ input features per drug. In addition, the average feature importance is shown (yellow dots). The per feature average was calculated based on the drugs for which this feature was selected in the feature selection.

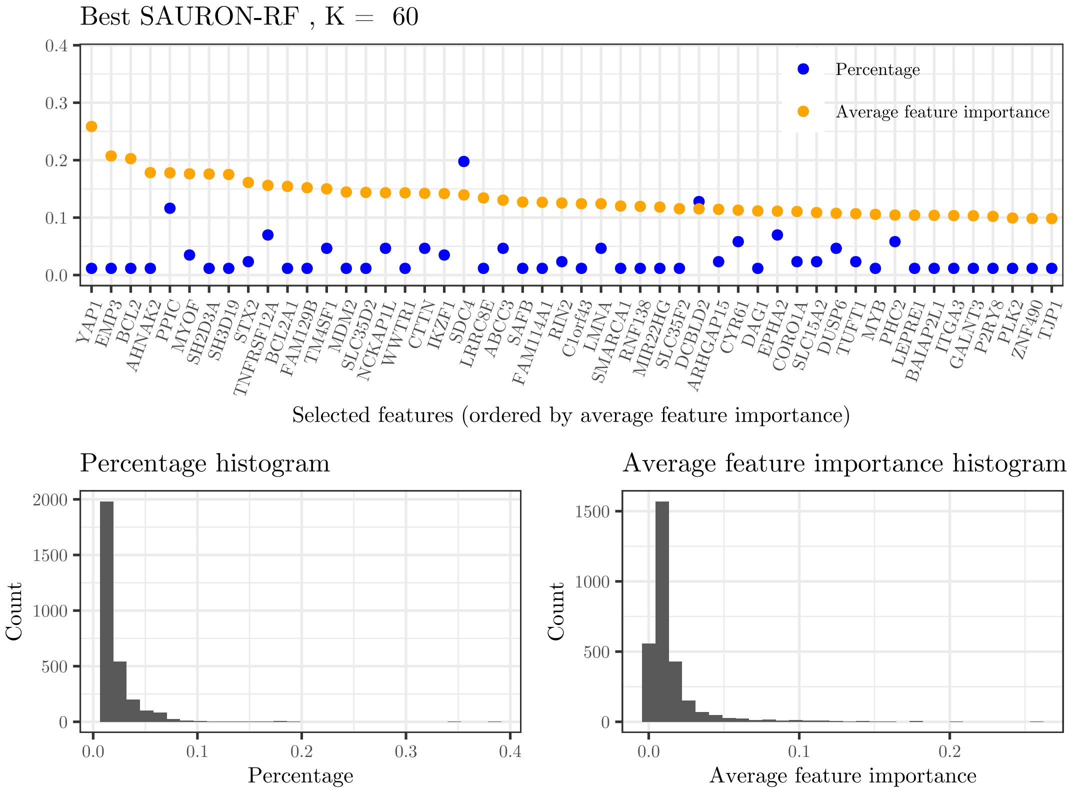

Figure 25: Feature importance for SAURON-RF using 60 input features (sorted by feature importance). This figure shows the 50 features with the highest average feature importance for the best-performing SAURON-RF model using $K = 60$ input features per drug. The per feature average was calculated based on the drugs for which this feature was selected in the feature selection. The corresponding percentage of drugs is also depicted (blue dots).

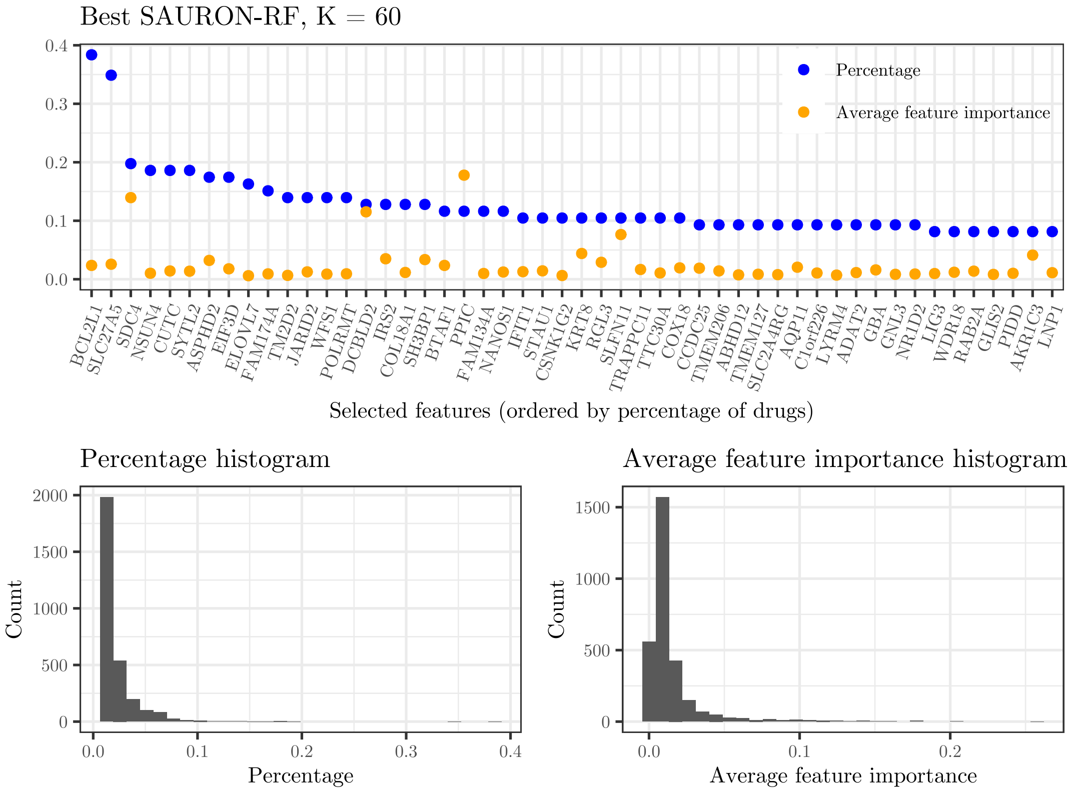

Figure 26: Feature importance for SAURON-RF using 60 input features (sorted by percentage of drugs affected). This figure shows the features sorted decreasingly by the percentage of drugs for which they were selected when using $K = 60$ input features per drug. In addition, the average feature importance is shown (yellow dots). The per feature average was calculated based on the drugs for which this feature was selected in the feature selection.

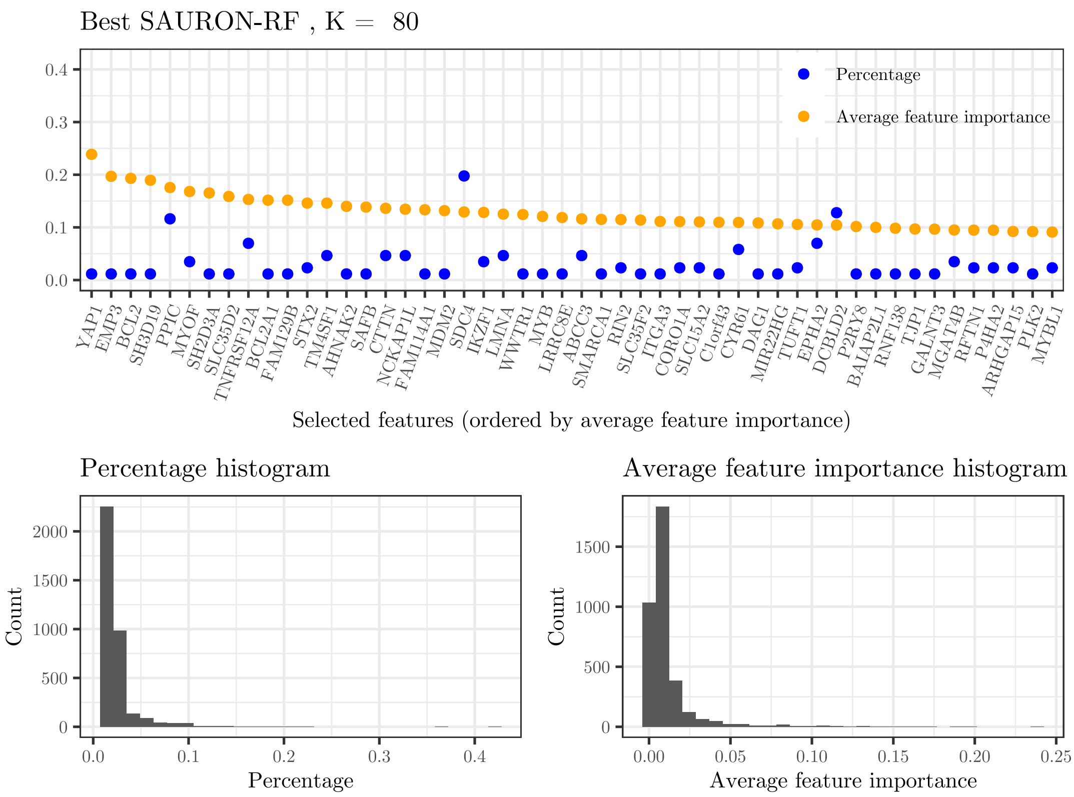

Figure 27: Feature importance for SAURON-RF using 80 input features (sorted by feature importance). This figure shows the 50 features with the highest average feature importance for the best-performing SAURON-RF model using $K = 80$ input features per drug. The per feature average was calculated based on the drugs for which this feature was selected in the feature selection. The corresponding percentage of drugs is also depicted (blue dots).

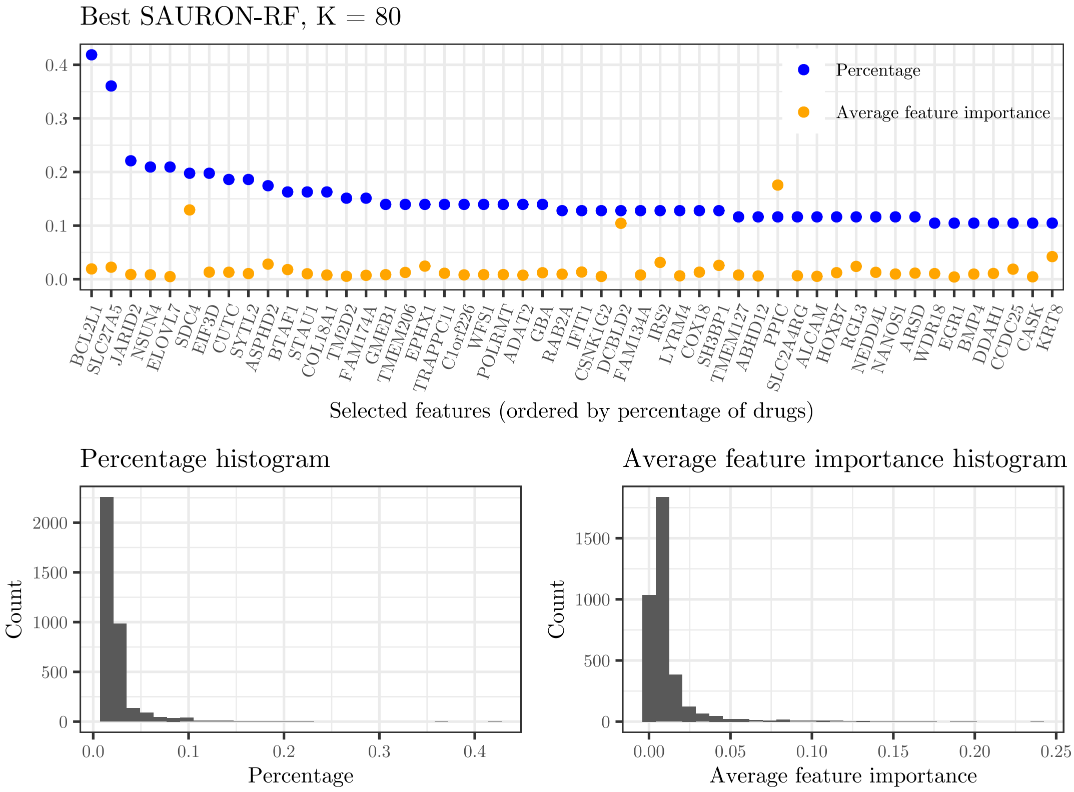

Figure 28: Feature importance for SAURON-RF using 80 input features (sorted by percentage of drugs affected). This figure shows the features sorted decreasingly by the percentage of drugs for which they were selected when using $K = 80$ input features per drug. In addition, the average feature importance is shown (yellow dots). The per feature average was calculated based on the drugs for which this feature was selected in the feature selection.

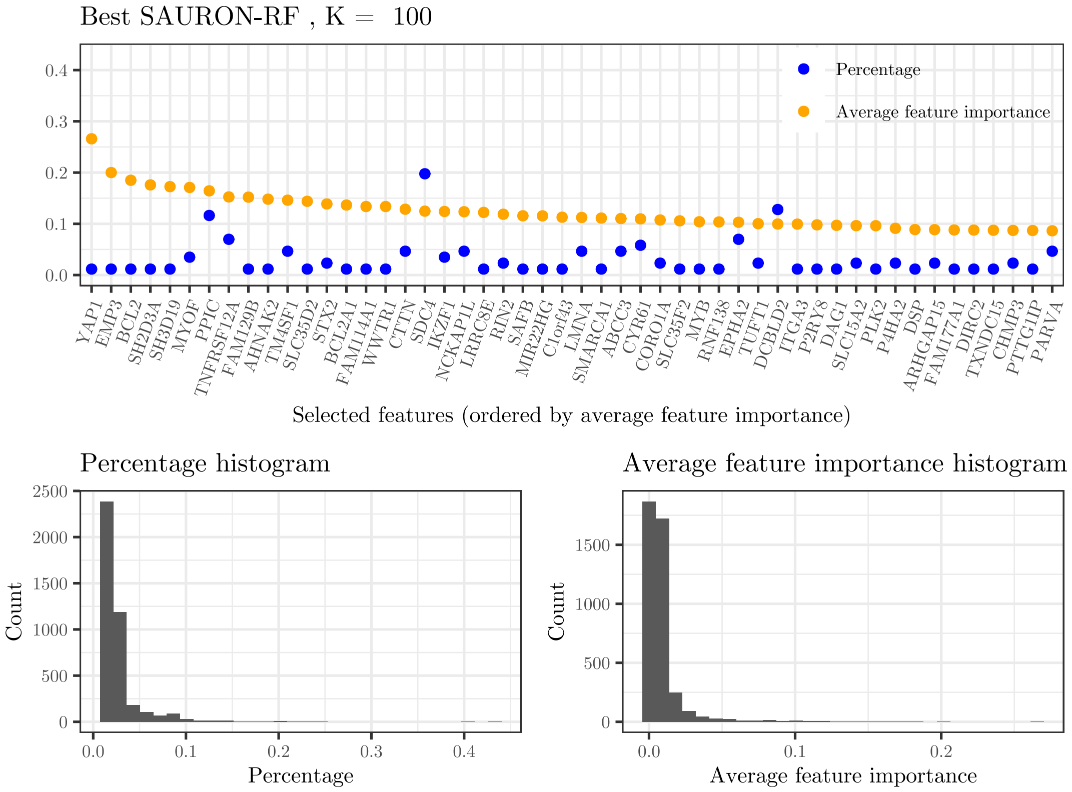

Figure 29: Feature importance for SAURON-RF using 100 input features (sorted by feature importance). This figure shows the 50 features with the highest average feature importance for the best-performing SAURON-RF model using $K = 100$ input features per drug. The per feature average was calculated based on the drugs for which this feature was selected in the feature selection. The corresponding percentage of drugs is also depicted (blue dots).

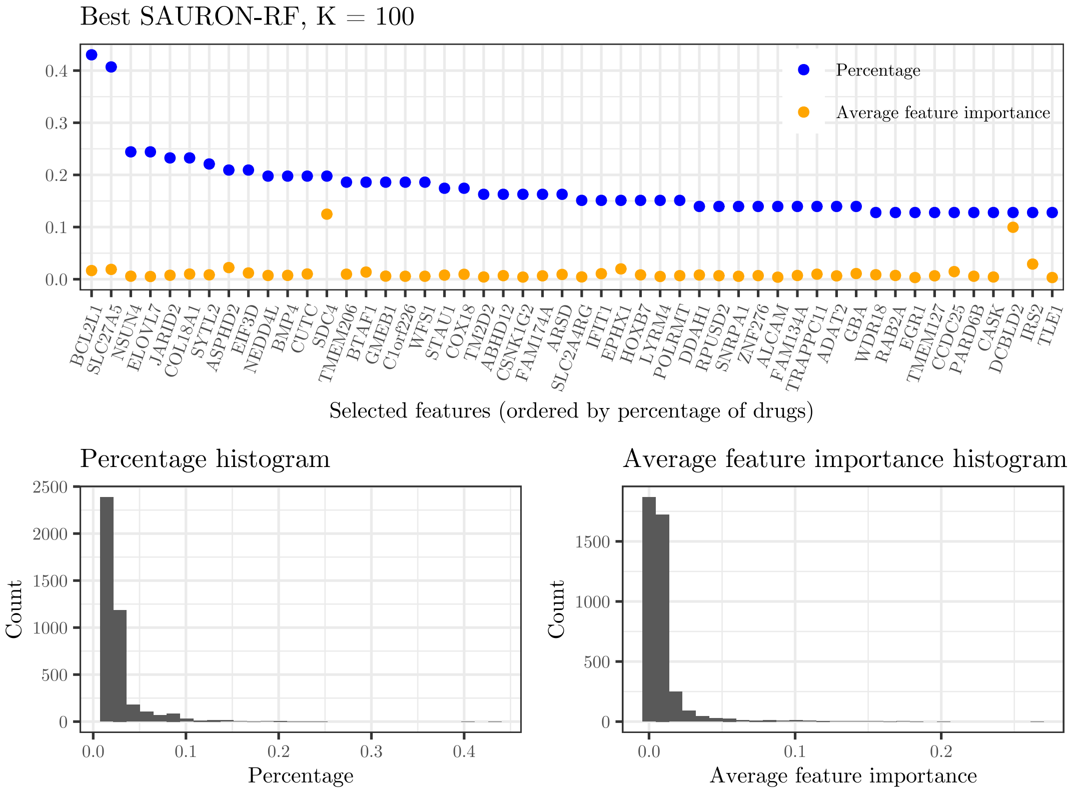

Figure 30: Feature importance for SAURON-RF using 100 input features (sorted by percentage of drugs affected). This figure shows the features sorted decreasingly by the percentage of drugs for which they were selected when using $K = 100$ input features per drug. In addition, the average feature importance is shown (yellow dots). The per feature average was calculated based on the drugs for which this feature was selected in the feature selection.

References

Kwak, N., et al. ”Input feature selection for classification problems” IEEE transactions on neural networks 13.1 (2002): 143 - 159. https://doi.org/10.1109/72.977291

Whitsett, Timothy G., et al. ”Mcl-1 mediates TWEAK/Fn14-induced non–small cell lung cancer survival and therapeutic response.” Molecular Cancer Research 12.4 (2014): 550-559. https://doi.org/10.1158/1541-7786.mcr-13-0458

Pasqualini, Lorenza, et al. ”miR-22 and miR-29a are members of the androgen receptor cistrome modulating LAMC1 and Mcl-1 in prostate cancer.” Molecular endocrinology 29.7 (2015): 1037-1054. https://doi.org/10.1210/me.2014-1358

Zhang, Le, et al. ”Emerging impact of the long noncoding RNA MIR22HG on proliferation and apoptosis in multiple human cancers” Journal of Experimental & Clinical Cancer Research 39.1 (2020): 1-12. https://doi.org/10.1186/s13046-020-01784-8

Rees, M. G., et al. ”Correlating chemical sensitivity and basal gene expression reveals mechanism of action” Nature chemical biology 12.2 (2016): 109-116. https://doi.org/10.1038/nchembio.1986

Chan, Steven M., et al. ”Isocitrate dehydrogenase 1 and 2 mutations induce BCL-2 dependence in acute myeloid leukemia.” Nature medicine 21.2 (2015): 178-184. https://doi.org/10.1038/nm.3788

Rahmani, M., et al. ”Co-administration of the mTORC1/TORC2 inhibitor INK128 and the Bcl-2/Bcl-xL antagonist ABT-737 kills human myeloid leukemia cells through Mcl-1 down-regulation and AKT inactivation” Haematologica 100.12 (2015): 1553-1563. https://doi.org/10.3324/haematol.2015.130351

Pishas, Kathleen I., et al. ”Nutlin-3a efficacy in sarcoma predicted by transcriptomic and epigenetic profiling.” Cancer research 74.3 (2014): 921-931. https://doi.org/10.1158/0008-5472.can-13-2424

Stoyanova, Tanya, et al. ”DDB2 decides cell fate following DNA damage.” Proceedings of the National Academy of Sciences 106.26 (2009): 10690-10695. https://doi.org/10.1073/pnas.0812254106

Zanjirband, M., et al. ”Pre-clinical efficacy and synergistic potential of the MDM2-p53 antagonists, Nutlin-3 and RG7388, as single agents and in combined treatment with cisplatin in ovarian cancer” Oncotarget 7.26 (2016): 40115-40134. https://doi.org/10.18632/oncotarget.9499

Kumamoto, K., et al. ”Nutlin-3a activates p53 to both down-regulate inhibitor of growth 2 and up-regulate mir-34a, mir-34b, and mir-34c expression, and induce senescence” Cancer research 68.9 (2008): 3193-3203. https://doi.org/10.1158/0008-5472.can-07-2780

Coussy, Florence, et al. "BRCAness, SLFN11, and RB1 loss predict response to topoisomerase I inhibitors in triple-negative breast cancers." Science Translational Medicine 12.531 (2020): eaax2625. https://doi.org/10.1126/scitranslmed.aax2625

Krushkal, J., et al. "Longitudinal transcriptional response of glycosylation-related genes, regulators, and targets in cancer cell lines treated with 11 antitumor agents" Cancer Informatics 16 (2017): 1176935117747259. https://doi.org/10.1177/1176935117747259

Xing, S., et al. "WGCNA reveals key gene modules regulated by the combined treatment of colon cancer with PHY906 and CPT11" Bioscience Reports 40.9 (2020). https://doi.org/10.1042/bsr20200935

Alam, Manzar, et al. "Bax/Bcl-2 Cascade is Regulated by EGFR Pathway: Therapeutic Targeting of Non-Small Cell Lung Cancer." Frontiers in Oncology (2022): 933. https://doi.org/10.3389/fonc.2022.869672

Ge, X., et al. "Hypoxia-mediated mitochondria apoptosis inhibition induces temozolomide treatment resistance through miR-26a/Bad/Bax axis" Cell Death & Disease 9.11 (2018): 1-16. https://doi.org/10.1038/s41419-018-1176-7

Wu, Chiao-En, et al. "ATM dependent DUSP6 modulation of p53 involved in synergistic targeting of MAPK and p53 pathways with trametinib and MDM2 inhibitors in cutaneous melanoma." Cancers 11.1 (2018): 3. https://doi.org/10.3390/cancers11010003

Da Vià, Matteo Claudio, et al. "CIC mutation as a molecular mechanism of acquired resistance to combined BRAF-MEK inhibition in extramedullary multiple myeloma with central nervous system involvement." The oncologist 25.2 (2020): 112-118. https://doi.org/10.1634/theoncologist.2019-0356

Flaherty, Keith T. "Moving Forward: Making BRAF-Targeted Therapy Better." BRAF Targets in Melanoma. Springer, New York, NY, 2015. 183-201. https://doi.org/10.1007/978-1-4939-2143-0_9

Eriksson, Johanna, et al. "Gene expression analyses of primary melanomas reveal CTHRC1 as an important player in melanoma progression." Oncotarget 7.12 (2016): 15065. https://doi.org/10.18632/oncotarget.7604

Li, J. X., et al. "The B-RafV600E inhibitor dabrafenib selectively inhibits RIP3 and alleviates acetaminophen-induced liver injury." Cell death & disease 5.6 (2014): e1278-e1278. https://doi.org/10.1038/cddis.2014.241

Santini, Cristina C., et al. "Global view of the RAF-MEK-ERK module and its immediate downstream effectors." Scientific reports 9.1 (2019): 1-11. https://doi.org/10.1038/s41598-019-47245-x

Tan, Zhigang, Jizong Zhao, and Yugang Jiang. "MiR-634 sensitizes glioma cells to temozolomide by targeting CYR 61 through Raf-ERK signaling pathway." Cancer Medicine 7.3 (2018): 913-921. https://doi.org/10.1002/cam4.1351

Iacobucci, Ilaria, et al. "IKAROS deletions dictate a unique gene expression signature in patients with adult B-cell acute lymphoblastic leukemia." PloS one 7.7 (2012): e40934. https://doi.org/10.1371/journal.pone.0040934

Schreck, Karisa C., et al. "Combination MEK and mTOR inhibitor therapy is active in models of glioblastoma." Neuro-oncology advances 2.1 (2020): vdaa138. https://doi.org/10.1093/noajnl/vdaa138

Cheng, Shuai, et al. "Transmembrane protein DCBLD2 is correlated with poor prognosis and affects phenotype by regulating epithelial-mesenchymal transition in human glioblastoma cells" NeuroReport 32.6 (2021): 507-517. https://doi.org/10.1097/wnr.0000000000001611

He, Jie, et al. "Association of DCBLD2 upregulation with tumor progression and poor survival in colorectal cancer" Cellular Oncology 43.3 (2020): 409-420. https://doi.org/10.1007/s13402-020-00495-8

Xie, Pan, et al. "DCBLD2 Affects the Development of Colorectal Cancer via EMT and Angiogenesis and Modulates 5-FU Drug Resistance" Frontiers in Cell and Developmental Biology 9 (2021). https://doi.org/10.3389/fcell.2021.669285

Kumawat, Manoj, et al. "Salmonella Typhimurium peptidyl-prolyl cis–trans isomerase C (PPIase C) plays a substantial role in protein folding to maintain the protein structure" World Journal of Microbiology and Biotechnology 36.11 (2020): 1-7. https://doi.org/10.1007/s11274-020-02943-x

Elfenbein, Arye, and Simons, Michael. "Syndecan-4 signaling at a glance." Journal of cell science 126.17 (2013): 3799-3804. https://doi.org/10.1242/jcs.124636

Safran, Marilyn, et al. "GeneCards Version 3: the human gene integrator." Database 2010 (2010). https://doi.org/10.1093/database/baq020
